# Effects of prophylactic ibuprofen and paracetamol administration on the immunogenicity and reactogenicity of the 10-valent pneumococcal non-typeable *Haemophilus influenzae* protein D conjugated vaccine (PHiD-CV) co-administered with DTPa-combined vaccines in children: An open-label, randomized, controlled, non-inferiority trial

**DOI:** 10.1080/21645515.2016.1223001

**Published:** 2016-08-19

**Authors:** Oana Falup-Pecurariu, Sorin C. Man, Mihai L. Neamtu, Gratiana Chicin, Ginel Baciu, Carmen Pitic, Alexandra C. Cara, Andrea E. Neculau, Marin Burlea, Ileana L. Brinza, Cristina N. Schnell, Valentina Sas, Valeriu V. Lupu, Nancy François, Kristien Swinnen, Dorota Borys

**Affiliations:** a Department of Pediatrics, Children's Clinic Hospital, Faculty of Medicine, Transilvania University, Brasov, Romania; b Mother and Child Department, University of Medicine and Pharmacy “Iuliu Hatieganu,” Cluj-Napoca, Romania; c Pediatric Clinic, Pediatric Clinic Hospital Sibiu, Sibiu, Romania; d Medical Department, Lucian Blaga University of Sibiu, Sibiu, Romania; e Preventive Medicine Department, Prophylaxis Center, Timisoara, Romania; f Department of Pediatrics, Dunarea de Jos University of Galati, Galati, Romania; g Saint Andrew Children Hospital Galati, Galati, Romania; h General Practitioner, Private Practice, Galati, Romania; i General Practitioner, Private Practice, Calarasi, Romania; j Fundamental and Prophylactic Sciences Department, Transilvania University, Brasov, Romania; k Department of Pediatrics, Grigore T. Popa University of Medicine and Pharmacy, Iasi, Romania; l General Practitioner, Private Practice, Braila, Romania; m Third Pediatric Clinic, Emergency Clinical Hospital for Children, Cluj-Napoca, Romania; n GSK, Wavre, Belgium; o XPE Pharma & Science for GSK, Wavre, Belgium

**Keywords:** fever, Ibuprofen, paracetamol, prophylaxis, vaccine, 10-valent pneumococcal conjugate

## Abstract

Prophylactic paracetamol administration impacts vaccine immune response; this study (www.clinicaltrials.gov: NCT01235949) is the first to assess PHiD-CV immunogenicity following prophylactic ibuprofen administration. In this phase IV, multicenter, open-label, randomized, controlled, non-inferiority study in Romania (November 2010–December 2012), healthy infants were randomized 3:3:3:1:1:1 to prophylactically receive immediate, delayed or no ibuprofen (IIBU, DIBU, NIBU) or paracetamol (IPARA, DPARA, NPARA) after each of 3 primary doses (PHiD-CV at age 3/4/5 months co-administered with DTPa-HBV-IPV/Hib at 3/5 and DTPa-IPV/Hib at 4 months) or booster dose (PHiD-CV and DTPa-HBV-IPV/Hib; 12–15 months). Non-inferiority of immune response one month post-primary vaccination in terms of percentage of infants with anti-pneumococcal antibody concentrations ≥0.2 µg/mL (primary objective) was demonstrated if the upper limit (UL) of the 98.25% confidence interval of difference between groups (NIBU vs IIBU, NIBU vs DIBU) was <10% for ≥7/10 serotypes. Immunogenicity and reactogenicity/safety were evaluated, including confirmatory analysis of difference in fever incidences post-primary vaccination in IBU or DIBU group compared to NIBU. Of 850 infants randomized, 812 were included in the total vaccinated cohort. Non-inferiority was demonstrated for both comparisons (UL was <10% for 9/10 vaccine serotypes; exceptions: 6B [NIBU], 23F [IIBU]). However, fever incidence post-primary vaccination in the IIBU and DIBU groups did not indicate a statistically significant reduction. Prophylactic administration (immediate or delayed) of paracetamol decreased fever incidence but seemed to reduce immune response to PHiD-CV, except when given only at booster. Twenty-seven serious adverse events were reported for 15 children; all resolved and were not vaccination-related.

## Introduction

Pneumococcal conjugate vaccines (PCVs) provide protection against invasive pneumococcal disease (IPD) and other diseases such as acute otitis media or pneumonia;^1-4^ PCVs have been included in many national childhood immunization programs.

Co-administration of PCVs with standard infant vaccines was shown to induce a higher incidence of fever in children compared to single-vaccine administration.^5-7^ Antipyretics, most commonly paracetamol and ibuprofen, are sometimes administered prophylactically to prevent fever during pediatric immunization.^8^ Although prophylactic paracetamol administration significantly decreases febrile reactions, it has also been shown to reduce immune responses to some vaccine antigens.^9^ Prophylactic paracetamol administration (immediate and 6–8 hours post-vaccination) with 10-valent pneumococcal non-typeable *Haemophilus influenzae* protein D conjugate vaccine (PHiD-CV) transiently lowered immune response after primary and booster vaccination. Induction of immunological memory and persistent impact of PHiD-CV on carriage rates were observed until at least 28 months post-booster vaccination.^10^ The observed trend toward lower antibody geometric mean concentrations (GMCs) prior to boosting may have significance for those children who might miss their booster dose, as their antibodies may decline faster than if they had not received paracetamol. Prophylactic administration of paracetamol also seemed to interfere with immune responses to the PCV13 in infants, while ibuprofen appeared to reduce responses to pertussis filamentous haemagglutinin (FHA) and tetanus antigens without impacting pneumococcal responses.^11^

In contrast to these data, a recent study showed that prophylactic administration of paracetamol in children after concomitant vaccination with a multicomponent meningococcal serogroup B vaccine (4CMenB), DTPa-HBV-IPV/Hib and PCV7 decreased fever and reactogenicity, with no apparent clinically relevant effect on immune responses.^12^

To date, there are no published data concerning the impact of prophylactic ibuprofen administration on the immune response to PHiD-CV.^13^ This study aimed to demonstrate non-inferiority of the immune response to PHiD-CV administered as a 3-dose primary course with immediate (IIBU) or delayed (DIBU) versus no prophylactic ibuprofen (NIBU) administration, in terms of percentage of infants with anti-pneumococcal antibody concentrations ≥0.2 µg/mL. Non-inferiority was to be demonstrated if, for ≥7/10 serotypes, the upper limit (UL) of the 98.25% confidence interval (CI) of the difference between groups (NIBU vs IIBU and NIBU vs DIBU) was <10%, in compliance with the European Medicines Agency Guideline on the Choice of the Non-inferiority Margin.^14^ Additionally, the study aimed to demonstrate a lower incidence of febrile reactions with immediate or delayed ibuprofen administration vs no ibuprofen administration.

We also assessed the effect of paracetamol administration (immediate or delayed, the latter not yet studied) on the immunogenicity and reactogenicity of PHiD-CV and the co-administered routine infant vaccines after primary and booster vaccinations. With this information, clinicians can objectively assess if the benefits of prophylaxis of febrile reactions outweigh the risk of potential effects on immunization.

## Results

### Study participants

**Figure 1.**
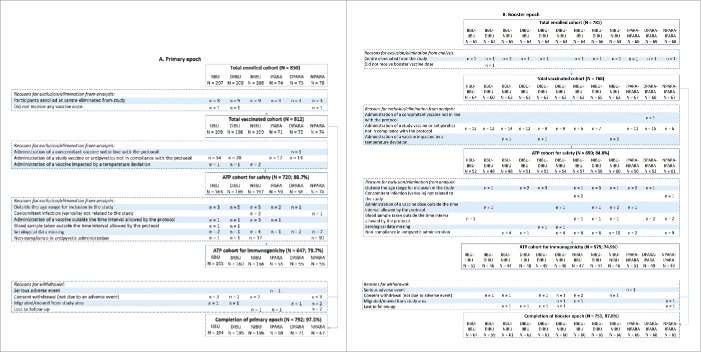
Participant flow chart. Footnote: **Primary vaccination**: PHiD-CV and DTPa-(HBV)-IPV/Hib at 3, 4, and 5 months of age, with the following prophylactic antipyretic regimen: IIBU, immediate ibuprofen; DIBU, delayed ibuprofen; NIBU, no ibuprofen; IPARA, immediate paracetamol; DPARA, delayed paracetamol; NPARA, no paracetamol. **Booster vaccination**: PHiD-CV and DTPa-HBV-IPV/Hib at 12–15 months of age, with the following prophylactic antipyretic regimen: at primary vaccination: immediate ibuprofen, and at booster: immediate (IIBU-IIBU), delayed (IIBU-DIBU) or no ibuprofen (IIBU-NIBU); at primary vaccination: delayed ibuprofen, and at booster: immediate (DIBU-IIBU), delayed (DIBU-DIBU) or no ibuprofen (DIBU-NIBU); at primary vaccination: no ibuprofen, and at booster: immediate (NIBU-IIBU), delayed (NIBU-DIBU) or no ibuprofen (NIBU-NIBU); immediate paracetamol at primary vaccination and no paracetamol at booster (IPARA-NPARA); delayed paracetamol at primary vaccination and immediate paracetamol at booster (DPARA-IPARA); no paracetamol at primary vaccination, and immediate paracetamol at booster (NPARA-IPARA). ATP, according-to-protocol; N, number of participants.

The study was conducted between 12 November 2010 and 08 December 2012. Of 850 participants randomized, 812 were included in the total vaccination cohort (TVC) for primary vaccination and 768 in the TVC for booster vaccination (Fig. 1); 647 (79.7%) children from the primary and 575 (74.9%) children from the booster epoch were included in the according-to-protocol (ATP) cohort for immunogenicity. Demographic characteristics were similar between groups (Table S1). The mean age at primary vaccination was 13.1 (standard deviation: 1.18) weeks at first dose, 18.0 (1.48) weeks at second dose, and 23.1 (1.78) weeks at third dose; the mean age at booster vaccination was 12.3 (0.62) months. There were no major differences between groups in the total daily dose of administered antipyretics. Two children in the TVC were withdrawn due to a serious adverse event (SAE) during the study period; these SAEs were not considered to be causally related to vaccination.

### Effect of ibuprofen on PHiD-CV immunogenicity

One month post-primary vaccination, for each of the 10 vaccine serotypes, the percentage of children in the IBU groups with antibody concentrations ≥0.2 µg/mL was at least 98.7%, except for serotypes 6B and 23F (6B at least 84.0%; 23F at least 89.2% in each group). Non-inferiority in terms of the percentage of infants with antibody concentrations ≥0.2 µg/mL was demonstrated since the UL of the difference was <10% for 9 out of 10 serotypes for each comparison (IIBU vs NIBU and DIBU vs NIBU). The UL was >10% for serotypes 6B (IIBU vs NIBU: percentage difference 0.69; UL = 10.99%) and 23F (DIBU vs NIBU: percentage difference 2.73; UL = 11.04%). No statistically significant differences in antibody GMCs for vaccine pneumococcal serotypes or protein D were observed (Table 1).

**Table 1.** Serotype-specific pneumococcal and protein D antibody responses with pairwise group comparisons for the ibuprofen groups, at one month post-dose three (ATP cohort for immunogenicity).

Proportion of children with antibody concentrations ≥ 0.2 µg/mL					
	% ≥ 0.2 µg/mL (95% CI)			Difference in % ≥ 0.2 µg/mL	
Serotype	IIBU N = 154	DIBU N = 158	NIBU N = 164	NIBU minus IIBU 98.25% CI (LL; UL)	NIBU minus DIBU 98.25% CI (LL; UL)
Vaccine serotypes					
**1**	100 (97.5; 100)	100 (97.6; 100)	99.4 (96.6; 100)	−0.62 (−4.52; 3.17)	−0.62 (−4.52; 2.91)
**4**	99.3 (96.2; 100)	100 (97.6; 100)	99.4 (96.5; 100)	0.06 (−3.94; 4.38)	−0.63 (−4.57; 2.91)
**5**	100 (97.5; 100)	100 (97.6; 100)	99.4 (96.5; 100)	−0.64 (−4.63; 3.19)	−0.64 (−4.63; 2.92)
**6B**	84.0 (77.0; 89.6)	87.1 (80.8; 91.9)	84.7 (78.1; 90.0)	0.69 (−9.40; **10.99**)	−2.38 (−12.02; 7.22)
**7F**	99.4 (96.4; 100)	100 (97.7; 100)	100 (97.8; 100)	0.65 (−2.70; 4.71)	0.00 (−3.34; 3.48)
**9V**	99.3 (96.2; 100)	100 (97.6; 100)	98.7 (95.5; 99.8)	−0.58 (−5.05; 3.82)	−1.27 (−5.66; 2.32)
**14**	100 (97.5; 100)	99.4 (96.4; 100)	99.4 (96.5; 100)	−0.65 (−4.68; 3.15)	0.00 (−4.08; 4.12)
**18C**	99.3 (96.2; 100)	99.4 (96.4; 100)	98.7 (95.5; 99.8)	−0.58 (−5.04; 3.85)	−0.62 (−5.08; 3.54)
**19F**	100 (97.5; 100)	98.7 (95.4; 99.8)	99.4 (96.5; 100)	−0.63 (−4.60; 3.14)	0.67 (−3.40; 5.20)
**23F**	91.9 (86.3; 95.7)	89.2 (83.3; 93.6)	92.0 (86.7; 95.7)	0.08 (−7.66; 8.10)	2.73 (−5.30; **11.04**)
Vaccine-related serotypes					
**6A**	44.2 (36.0; 52.6)	47.4 (39.2; 55.6)	43.3 (35.4; 51.4)	NA	NA
**19A**	53.1 (44.6; 61.4)	52.0 (43.7; 60.1)	40.1 (32.4; 48.2)	NA	NA
**Antibody GMCs**					
	**Antibody GMC (95% CI)**			**Antibody GMC ratio**	
**Serotype**	**IIBU N = 154**	**DIBU N = 158**	**NIBU N = 164**	**IIBU / NIBU 99.8% CI (LL; UL)**	**DIBU / NIBU 99.8% CI (LL; UL)**
Vaccine serotypes (µg/mL)					
**1**	1.82 (1.59; 2.09)	1.71 (1.49; 1.95)	1.90 (1.67; 2.17)	0.96 (0.71; 1.29)	0.90 (0.67; 1.21)
**4**	2.25 (1.97; 2.57)	2.21 (1.95; 2.51)	2.21 (1.96; 2.50)	1.02 (0.77; 1.35)	1.00 (0.76; 1.32)
**5**	2.93 (2.58; 3.33)	2.39 (2.13; 2.69)	2.77 (2.44; 3.15)	1.06 (0.80; 1.41)	0.86 (0.66; 1.14)
**6B**	0.67 (0.55; 0.81)	0.76 (0.63; 0.92)	0.60 (0.49; 0.72)	1.12 (0.72; 1.74)	1.28 (0.83; 1.97)
**7F**	2.87 (2.52; 3.27)	2.83 (2.52; 3.17)	2.77 (2.49; 3.09)	1.04 (0.79; 1.35)	1.02 (0.80; 1.31)
**9V**	2.10 (1.81; 2.42)	2.01 (1.79; 2.27)	2.18 (1.91; 2.50)	0.96 (0.70; 1.31)	0.92 (0.69; 1.22)
**14**	4.76 (4.10; 5.51)	4.52 (3.91; 5.21)	4.77 (4.08; 5.58)	1.00 (0.71; 1.40)	0.95 (0.68; 1.32)
**18C**	3.85 (3.23; 4.60)	3.80 (3.24; 4.46)	4.34 (3.65; 5.15)	0.89 (0.60; 1.31)	0.88 (0.60; 1.27)
**19F**	6.11 (5.26; 7.10)	5.04 (4.35; 5.86)	4.96 (4.22; 5.83)	1.23 (0.87; 1.75)	1.02 (0.72; 1.44)
**23F**	1.04 (0.86; 1.26)	0.92 (0.76; 1.11)	1.07 (0.91; 1.26)	0.97 (0.66; 1.44)	0.86 (0.58; 1.27)
Vaccine-related serotypes (µg/mL)					
**6A**	0.17 (0.14; 0.21)	0.18 (0.15; 0.23)	0.15 (0.12; 0.19)	NA	NA
**19A**	0.23 (0.18; 0.28)	0.20 (0.16; 0.25)	0.16 (0.13; 0.19)	NA	NA
Protein D (EL.U/mL)					
	1461.28 (1267.4; 1684.8)	1353.13 (1191.3; 1537.0)	1557.75 (1355.4; 1790.3)	0.94 (0.69; 1.28)	0.87 (0.64; 1.17)

Footnote: PHiD-CV and DTPa-(HBV)-IPV/Hib at 3, 4, and 5 months of age, with the following prophylactic antipyretic regimen: IIBU, immediate ibuprofen; DIBU, delayed ibuprofen; NIBU, no ibuprofen; N = maximum number of children with available results; LL, lower limit; UL, upper limit; CI, confidence interval; 98.25% CI, standardized asymptotic confidence interval; GMC, geometric mean antibody concentration; %, percentage of participants with anti-pneumococcal serotype-specific antibody concentrations ≥ 0.2 µg/mL; NA, not available; ATP, according-to-protocol. A statistically significant difference was defined as an UL ≥10% for the 98.25% CI of the difference between groups, or an UL <1 for the 99.8% CI of the GMC ratios (**bold**).

Post-booster immune responses were in similar ranges in all groups; for each of the vaccine serotypes, percentages of children with antibody concentrations ≥0.2 µg/mL were 91.5–100%. An increase in antibody GMCs post-booster compared to post-primary vaccination was observed in all groups, for each vaccine serotype except serotype 14 in the DIBU-NIBU group and serotype 19F in the NPARA-IPARA group (Table S2). Because very few participants per group had available results from the opsonophagocytic activity and poliomyelitis neutralization assays, these results could not be presented and interpreted.

For vaccine-related serotypes 6A and 19A, antibody concentrations ≥0.2 µg/mL were observed for ≥43.3% and ≥40.1% of children in each IBU group at one month post-primary vaccination (Table 1), and for ≥80.4% and ≥78.0% children post-booster, respectively (Table S2).

### Effect of paracetamol on PHiD-CV immunogenicity

Post-primary vaccination, the percentage of children with antibody concentrations ≥0.2 µg/mL generally tended to be lower in the immediate (IPARA) and delayed paracetamol (DPARA) groups than in the no-paracetamol (NPARA) group (however, the 95% CI of the differences included 0) and the highest difference in point estimates vs control NPARA was observed for serotype 6B (∼8% for IPARA and ∼14% for DPARA). Compared to the NPARA group, antibody GMCs were lower for 6 of the PHiD-CV serotypes and protein D in the IPARA group, and for serotypes 1 and 6B in the DPARA group (Table 2).

**Table 2.** Exploratory analysis: serotype-specific pneumococcal and protein D antibody responses with pairwise group comparisons for the paracetamol groups, one month post-dose three (ATP cohort for immunogenicity).

Proportion of children with antibody concentrations ≥ 0.2 µg/mL					
	% ≥ 0.2 µg/mL (95% CI)			Difference in % ≥ 0.2 µg/mL	
Serotype	IPARA N = 55	DPARA N = 55	NPARA N = 56	NPARA minus IPARA 95% CI (LL; UL)	NPARA minus DPARA 95% CI (LL; UL)
Vaccine serotypes					
**1**	96.3 (87.3; 99.5)	98.0 (89.6; 100)	100 (93.5; 100)	3.70 (−3.01; 12.59)	1.96 (−4.69; 10.37)
**4**	96.4 (87.5; 99.6)	100 (93.0; 100)	100 (93.6; 100)	3.64 (−2.96; 12.38)	0.00 (−6.48; 7.07)
**5**	100 (93.3; 100)	100 (92.9; 100)	100 (93.4; 100)	0.00 (−6.70; 6.82)	0.00 (−6.70; 7.20)
**6B**	79.2 (65.9; 89.2)	72.5 (58.3; 84.1)	87.3 (75.5; 94.7)	8.03 (−6.35; 22.68)	14.72 (−0.55; 30.18)
**7F**	100 (93.5; 100)	100 (93.5; 100)	100 (93.6; 100)	0.00 (−6.47; 6.58)	0.00 (−6.47; 6.58)
**9V**	100 (93.3; 100)	100 (92.9; 100)	98.1 (90.1; 100)	−1.85 (−9.83; 5.03)	−1.85 (−9.83; 5.41)
**14**	100 (93.3; 100)	100 (92.9; 100)	100 (93.3; 100)	0.00 (−6.82; 6.82)	0.00 (−6.82; 7.20)
**18C**	98.1 (89.9; 100)	100 (92.9; 100)	100 (93.4; 100)	1.89 (−4.88; 10.00)	0.00 (−6.70; 7.20)
**19F**	100 (93.3; 100)	100 (92.9; 100)	100 (93.4; 100)	0.00 (−6.70; 6.82)	0.00 (−6.70; 7.20)
**23F**	87.0 (75.1; 94.6)	81.1 (68.0; 90.6)	90.9 (80.0; 97.0)	3.87 (−8.61; 16.76)	9.78 (−3.55; 23.71)
Vaccine-related serotypes					
**6A**	35.8 (23.1; 50.2)	30.0 (17.9; 44.6)	49.1 (35.1; 63.2)	NA	NA
**19A**	41.5 (28.1; 55.9)	50.0 (35.5; 64.5)	56.6 (42.3; 70.2)	NA	NA
**Antibody GMCs**					
	**Antibody GMC (95% CI)**			**Antibody GMC ratio**	
**Serotype**	**IPARA N = 55**	**DPARA N = 55**	**NPARA N = 56**	**IPARA / NPARA 95% CI (LL ; UL)**	**DPARA / NPARA 95% CI (LL ; UL)**
Vaccine serotypes (µg/mL)					
**1**	1.32 (1.04; 1.67)	1.38 (1.09; 1.74)	1.95 (1.64; 2.32)	0.68 (0.51; **0.90**)	0.71 (0.53; **0.94**)
**4**	1.57 (1.21; 2.04)	1.95 (1.63; 2.32)	2.59 (2.07; 3.24)	0.61 (0.43; **0.85**)	0.75 (0.56; 1.00)
**5**	1.95 (1.53; 2.48)	2.36 (1.89; 2.94)	3.05 (2.53; 3.68)	0.64 (0.47; **0.86**)	0.77 (0.58; 1.03)
**6B**	0.49 (0.34; 0.69)	0.42 (0.28; 0.62)	0.72 (0.51; 1.02)	0.67 (0.41; 1.09)	0.58 (0.35; **0.97**)
**7F**	2.18 (1.75; 2.70)	2.45 (2.01; 2.99)	2.95 (2.37; 3.69)	0.74 (0.54; 1.00)	0.83 (0.62; 1.11)
**9V**	1.67 (1.30; 2.13)	1.82 (1.48; 2.23)	2.40 (1.87; 3.10)	0.69 (0.49; **0.98**)	0.76 (0.55; 1.05)
**14**	3.44 (2.55; 4.62)	4.12 (3.21; 5.29)	5.17 (4.20; 6.36)	0.66 (0.46; **0.95**)	0.80 (0.58; 1.10)
**18C**	3.08 (2.29; 4.15)	4.08 (3.15; 5.29)	4.96 (3.75; 6.55)	0.62 (0.42; **0.93**)	0.82 (0.57; 1.20)
**19F**	4.95 (3.74; 6.54)	5.20 (3.94; 6.85)	6.98 (5.48; 8.88)	0.71 (0.49; 1.02)	0.75 (0.52; 1.07)
**23F**	0.77 (0.54; 1.09)	0.74 (0.51; 1.08)	1.00 (0.73; 1.36)	0.77 (0.48; 1.22)	0.74 (0.46; 1.20)
Vaccine-related serotypes (µg/mL)					
**6A**	0.11 (0.08; 0.16)	0.12 (0.08; 0.17)	0.19 (0.13; 0.27)	NA	NA
**19A**	0.15 (0.11; 0.22)	0.17 (0.12; 0.25)	0.25 (0.17; 0.36)	NA	NA
Protein D (EL.U/mL)					
	1109.64 (876.9; 1404.1)	1348.55 (1048.2; 1734.9)	1667.91 (1401.9; 1984.4)	0.67 (0.50; **0.89**)	0.81 (0.60; 1.09)

Footnote: PHiD-CV and DTPa-(HBV)-IPV/Hib at 3, 4, and 5 months of age, with the following prophylactic antipyretic regimen: IPARA, immediate paracetamol; DPARA, delayed paracetamol; NPARA, no paracetamol; N = maximum number of children with available results; LL, lower limit; UL, upper limit; 95% CI, standardized asymptotic confidence interval; GMC, geometric mean antibody concentration; %, percentage of participants with anti-pneumococcal serotype-specific antibody concentrations ≥ 0.2 µg/mL; NA, not available; ATP, according-to-protocol. The exclusion of 0 from the 95% CI of difference between groups, and the exclusion of 1 from the 95% CI of antibody GMC ratios were used to highlight potential group differences (**bold**).

One month post-booster vaccination, for each of the 10 vaccine serotypes, at least 91.7%, 93.2% and 97.9% of children in the IPARA-NPARA, DPARA-IPARA and NPARA-IPARA groups, respectively, had antibody concentrations ≥0.2 µg/mL. Post-booster antibody GMCs tended to be lower than in the control NIBU-NIBU group for all vaccine serotypes in the IPARA-NPARA group and the majority of serotypes in the DPARA-IPARA group, as well as for protein D in both groups. No major differences in antibody GMCs were observed when paracetamol was administered only during booster vaccination (NPARA-IPARA group) (Table S3).

For vaccine-related serotypes 6A and 19A, antibody concentrations ≥0.2 µg/mL were observed for ≥30.0% and ≥41.5% of children in each PARA group at one month post-primary vaccination (Table 2), and for ≥83.0% and ≥77.3% children post-booster (Table S3).

### Effect of ibuprofen on co-administered antigens

Post-primary vaccination, a borderline significant difference in antibody GMCs was observed in the IIBU vs NIBU comparison for FHA (UL = 0.99), in the DIBU vs NIBU comparison for tetanus (UL = 1.00) and in the IIBU vs NIBU comparison for hepatitis B surface antigen (HBs) (UL = 1.01) (Table 3). Post-booster, a difference in pertussis antibody GMCs was observed in the IIBU-DIBU (anti-pertussis toxoid [PT], anti-pertactin [PRN] and anti-FHA antibody GMCs) and IIBU-NIBU (anti-PT antibody GMCs) groups (Table S4). Seroprotection and seropositivity rates were not affected.

**Table 3.** Exploratory analysis: antibody responses to DTPa-HBV-IPV/Hib antigens with pairwise group comparisons, one month post-dose three (ATP cohort for immunogenicity).

	Seroprotection/seropositivity rates (95% CI)			GMC (95% CI)			GMC ratio	
Antibody (cut-off /threshold)	IIBU N = 138	DIBU N = 150	NIBU N = 155	IIBU N = 138	DIBU N = 150	NIBU N = 155	IIBU / NIBU 95% CI (LL; UL)	DIBU / NIBU 95% CI (LL; UL)
**DIPHT (≥0.1 IU/mL)**	100 (97.3; 100)	100 (97.6; 100)	100 (97.6; 100)	3.33 (2.97; 3.73)	2.94 (2.65; 3.26)	3.13 (2.80; 3.50)	1.06 (0.91; 1.24)	0.94 (0.81; 1.09)
**TET (≥0.1 IU/mL)**	100 (97.3; 100)	100 (97.6; 100)	100 (97.6; 100)	3.75 (3.31; 4.25)	3.37 (3.04; 3.74)	3.96 (3.52; 4.46)	0.95 (0.80; 1.12)	0.85 (0.73; 1.00)
**PT (≥5 EL.U/mL)**	100 (97.3; 100)	100 (97.5; 100)	100 (97.5; 100)	59.1 (53.7; 65.1)	64.2 (58.7; 70.2)	65.0 (59.6; 71.0)	0.91 (0.80; 1.03)	0.99 (0.87; 1.12)
**FHA (≥5 EL.U/mL)**	100 (97.2; 100)	100 (97.5; 100)	100 (97.4; 100)	163.1 (147.8; 180.1)	171.6 (154.7; 190.4)	191.1 (171.1; 213.5)	0.85 (0.74; **0.99**)	0.90 (0.77; 1.04)
**PRN (5 EL.U/mL)**	99.3 (96.0; 100)	100 (97.6; 100)	100 (97.6; 100)	103.9 (91.5; 118.0)	114.3 (101.0; 129.3)	118.1 (103.9; 134.3)	0.88 (0.73; 1.05)	0.97 (0.81; 1.16)
**PRP (≥0.15 µg/mL)**	100 (97.3; 100)	99.3 (96.3; 100)	100 (97.6; 100)	3.99 (3.27; 4.88)	3.66 (3.07; 4.36)	4.51 (3.75; 5.42)	0.89 (0.68; 1.16)	0.81 (0.63; 1.04)
**HBs (≥10 mIU/mL)**	100 (96.7; 100)	99.2 (95.4; 100)	100 (97.0; 100)	911.85 (719.55; 1155.56)	1139.10 (902.93; 1437.04)	1245.07 (998.97; 1551.80)	0.73 (0.53; 1.01)	0.91 (0.67; 1.26)
	**IPARA N = 50**	**DPARA N = 49**	**NPARA N = 53**	**IPARA N = 52**	**DPARA N = 49**	**NPARA N = 53**	**IPARA / NPARA 95% CI (LL; UL)**	**DPARA / NPARA 95% CI (LL; UL)**
**DIPHT (≥0.1 IU/mL)**	100 (92.9; 100)	100 (92.7; 100)	100 (93.3; 100)	3.06 (2.57; 3.66)	2.89 (2.32; 3.60)	3.46 (2.91; 4.11)	0.89 (0.69; 1.13)	0.84 (0.64; 1.10)
**TET (≥0.1 IU/mL)**	100 (92.7; 100)	100 (92.7; 100)	100 (92.7; 100)	2.94 (2.43; 3.56)	3.06 (2.55; 3.68)	3.76 (3.11; 4.56)	0.78 (0.60; 1.02)	0.81 (0.62; 1.06)
**PT (≥5 EL.U/mL)**	100 (92.5; 100)	100 (92.1; 100)	100 (93.3; 100)	60.4 (51.4; 71.0)	63.1 (52.0; 76.6)	61.52 (53.1; 71.2)	0.98 (0.79; 1.22)	1.03 (0.81; 1.30)
**FHA (≥5 EL.U/mL)**	100 (92.5; 100)	100 (92.1; 100)	100 (93.2; 100)	171.1 (139.8; 209.2)	196.5 (163.1; 236.8)	168.9 (141.9; 201.0)	1.01 (0.78; 1.32)	1.16 (0.90; 1.50)
**PRN (≥5 EL.U/mL)**	100 (92.5; 100)	100 (92.6; 100)	100 (93.3; 100)	97.1 (76.7; 123.1)	106.2 (83.1; 135.7)	114.0 (95.6; 136.0)	0.85 (0.64; 1.14)	0.93 (0.69; 1.25)
**PRP (≥0.15 µg/mL)**	100 (93.2; 100)	100 (92.5; 100)	100 (93.3; 100)	3.29 (2.36; 4.58)	4.23 (3.03; 5.91)	5.01 (3.69; 6.79)	0.66 (0.42; 1.03)	0.84 (0.54; 1.32)
**HBs (≥10 mIU/mL)**	97.5 (86.8; 99.9)	97.2 (85.5; 99.9)	100 (92.0; 100)	934.65 (580.70; 1504.32)	674.25 (373.26; 1217.95)	1027.79 (719.24; 1468.70)	0.91 (0.51; 1.62)	0.66 (0.34; 1.26)

Footnote: DIPHT, Diphtheria; TET, Tetanus; PT, Pertussis Toxoid; FHA, Filamentous Haemagglutinin; PRN, Pertactin; PRP, Polyribosyl-ribitol Phosphate; HBs, Hepatitis B Surface; EL.U/mL, ELISA units/milliliter; mIU/mL, milli-international units/milliliter; PHiD-CV and DTPa-(HBV)-IPV/Hib at 3, 4, and 5 months of age, with the following prophylactic antipyretic regimen: IIBU, immediate ibuprofen; DIBU, delayed ibuprofen; NIBU, no ibuprofen; IPARA, immediate paracetamol; DPARA, delayed paracetamol; NPARA, no paracetamol; N, maximum number of children with available results; LL, lower limit; UL, upper limit; 95% CI, confidence interval for the GMC ratio (Anova model – pooled variance); GMC, geometric mean concentration. The exclusion of 1 from the 95% CI of antibody GMC ratios was used to highlight potential group differences (**bold**).

### Effect of paracetamol on co-administered antigens

Concerning the co-administered vaccine antigens for which the results were interpretable (diphtheria, tetanus, pertussis [PT, FHA, and PRN], HBs and *Haemophilus influenzae* type b polyribosylribitol phosphate [PRP]), antibody GMCs seemed to be reduced in the IPARA and DPARA groups and in the NPARA-IPARA group for some antigens; nevertheless, seroprotection/seropositivity rates remained high (≥95.5%) (Table 3, Table S5). In detail, the antibody GMCs tended to be lower for post-primary anti-PRP (ratio of 0.66) and anti-tetanus (ratio of 0.78) in the IPARA group and for post-primary anti-tetanus (0.81) in the DPARA group (Table 3), as well as for post-booster anti-PT in the NPARA-IPARA and IPARA-NPARA groups compared to the control group (Table S5).

### Factorial design analysis

Comparison of antibody GMCs in the 9 booster groups that received ibuprofen did not indicate a combined effect (interaction) of prophylactic ibuprofen administration at primary and booster vaccination, and no individual effect of ibuprofen at primary vaccination or at booster on the PHiD-CV post-booster immune response.

### Safety results

**Figure 2.**
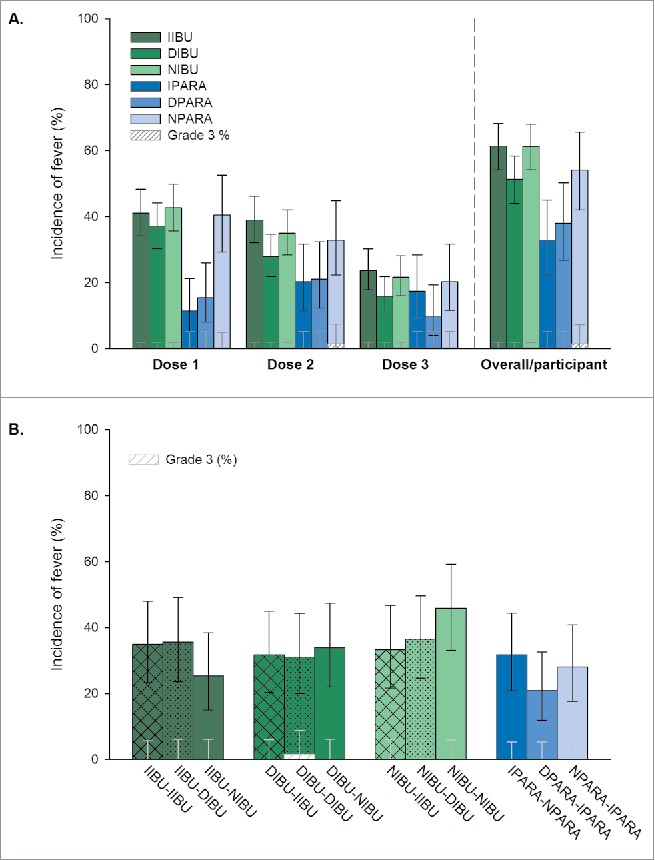
Incidence of fever post-primary (A) and post-booster (B) vaccination (TVC). Footnote: **Primary vaccination**: PHiD-CV and DTPa-(HBV)-IPV/Hib at 3, 4, and 5 months of age, with the following prophylactic antipyretic regimen: IIBU, immediate ibuprofen; DIBU, delayed ibuprofen; NIBU, no ibuprofen; IPARA, immediate paracetamol; DPARA, delayed paracetamol; NPARA, no paracetamol. **Booster vaccination**: PHiD-CV and DTPa-HBV-IPV/Hib at 12–15 months of age, with the following prophylactic antipyretic regimen: at primary vaccination: immediate ibuprofen, and at booster: immediate (IIBU-IIBU), delayed (IIBU-DIBU) or no ibuprofen (IIBU-NIBU); at primary vaccination: delayed ibuprofen, and at booster: immediate (DIBU-IIBU), delayed (DIBU-DIBU) or no ibuprofen (DIBU-NIBU); at primary vaccination: no ibuprofen, and at booster: immediate (NIBU-IIBU), delayed (NIBU-DIBU) or no ibuprofen (NIBU-NIBU); immediate paracetamol at primary vaccination and no paracetamol at booster (IPARA-NPARA); delayed paracetamol at primary vaccination and immediate paracetamol at booster (DPARA-IPARA); no paracetamol at primary vaccination, and immediate paracetamol at booster (NPARA-IPARA). Fever: rectal temperature ≥38.0°C; Grade 3 fever: rectal temperature >40°C or axillary/oral/tympanic temperature >39.5°C; TVC, total vaccinated cohort. Error bars indicate 95% confidence intervals.

The confirmatory analysis of the difference in fever incidences in the IIBU or DIBU groups compared to NIBU did not demonstrate any statistically significant reduction. Fever during primary vaccination was reported for 122 children (61.3%) in the NIBU group, compared to 121 (61.4%) in the IIBU group (difference: −0.11% [97.5% CI: −11.04; 10.82]) and 101 (51.3%) in the DIBU group (difference: 10.04% [97.5% CI: −1.15; 20.98]). Grade 3 fever was reported only in the NPARA and DIBU-DIBU group (Fig. 2).

Similar results were obtained in the complementary descriptive analysis on the ATP cohort: fever incidence in the NIBU group was 61.4%, vs 62.6% in the IIBU group (group difference: −1.16% [97.5% CI: −12.55; 10.39]) and 49.7% in the DIBU group (group difference: 11.72% [97.5% CI: 0.04; 23.13]).

Exploratory analyses indicated a trend for decrease in the rates of reported fever post-primary vaccination in the groups receiving immediate or delayed paracetamol (32.9% and 38.0% of participants, respectively) versus the control group (54.1% of participants) (Fig. 2).

Other solicited local and general symptoms seemed to be reported in similar ranges across groups during primary and booster vaccinations (Figures S1 and S2).

Twenty-seven SAEs were reported for a total of 15 children from the TVC. All SAEs resolved and none were considered by the investigator to be causally related to vaccination. In addition, of the 35 children at the study site eliminated from the TVC, 4 children reported SAEs including one fatal SAE (DPARA group; craniocerebral injury 132 d post-dose 3); none of these SAEs were considered by the investigator to be causally related to vaccination.

## Discussion

This study found no clinically relevant impact of immediate or delayed prophylactic administration of ibuprofen during primary or booster vaccination on the immune response to PHiD-CV. A factorial design analysis indicated neither a combined effect (interaction), nor separate effects of prophylactic ibuprofen administration at primary and booster vaccination on the post-booster immune response to PHiD-CV.

For the primary objective, a threshold of 0.2 µg/mL anti-pneumococcal antibody concentrations was used (equivalent to 0.35 µg/mL measured by the non-22F ELISA of the WHO reference laboratory). For most vaccine serotypes, almost all children in each study group (≥96.3%) reached this antibody concentration at 1 month post-primary vaccination, except for serotypes 6B (≥72.5%) and 23F (≥81.1%).

Prophylactic administration of paracetamol during primary series showed a trend for reduced post-primary anti-pneumococcal antibody GMCs when given immediately after vaccine administration (for the majority of vaccine serotypes) or when given in a delayed manner (for some serotypes). The proportion of children with post-primary antibody concentrations ≥0.2 µg/mL for PHiD-CV serotypes was not impacted except for serotypes 6B and 23F, thus the clinical relevance remains unknown. Observations related to immediate administration of paracetamol are in line with previous findings.^9^

When paracetamol was given immediately only at the booster dose, corresponding to the age with highest risk of febrile seizures,^15^ we observed no effect on immune response to PHiD-CV while fever was reduced. This suggests that paracetamol can be used for prophylaxis of febrile reactions at booster dose. In contrast, the post-booster immune response to PHiD-CV appeared to be impacted when paracetamol was administered either immediately at primary vaccination but not post-booster, or in a delayed manner at primary vaccination and immediately at booster dose.

Descriptive comparisons of the response to co-administered antigens showed a trend for lower anti-FHA post-primary antibody GMCs in the group with immediate prophylactic ibuprofen administration. No major differences were observed in post-booster antibody GMCs in the ibuprofen groups except for pertussis antigens (PT, FHA, and PRN in the IIBU-DIBU group, and PT in the IIBU-NIBU group vs the NIBU-NIBU group). However, seroprotection and seropositivity rates for the co-administered antigens one month after primary vaccination and one month after booster dose were not affected by ibuprofen prophylactic administration, suggesting no clinically relevant impact.

Our findings differ from *in vitro* assessments, in which ibuprofen was found to have a dose-dependent effect on antibody production in human peripheral blood mononuclear cells and in purified B cells, with the major influence on antibody production observed when ibuprofen was administered early (day 2 and 3) to the culture.^16^

Immediate or delayed prophylactic administration of paracetamol during primary vaccination did not reveal major differences in seroprotection and seropositivity rates or in antibody GMCs of co-administered antigens. When no antipyretics were given at booster dose, a trend for decreased post-booster antibody GMCs was observed for the majority of co-administered vaccine antigens, with no impact on seroprotection and seropositivity rates, indicating no or limited clinical relevance.

A previous study assessing the effect of prophylactic immediate administration of paracetamol at the time of vaccination with PHiD-CV and DTPa-HBV-IPV/Hib found generally lower antibody GMCs for antibodies against diphtheria, tetanus, PRN and PRP antigens after primary vaccination.^9^ After booster vaccination, this tendency was only observed for antibodies against tetanus. Moreover, this study showed that the seropositivity or seroprotection rates were not impacted and remained in line with previous experiences with DTPa-based or pneumococcal vaccines with the exception of serotype 6B after primary vaccination.^9^

Our results correspond with findings recently reported for PCV13, in which immediate prophylactic paracetamol administration seemed to interfere with infant series immune response to PCV13, while immediate prophylactic administration of ibuprofen did not interfere with pneumococcal responses but may reduce responses to pertussis FHA and tetanus antigens. These effects were especially apparent when antipyretic prophylaxis was administered at the time of primary vaccination, while no differences were observed after the booster dose.^11^

In contrast, another recent study did not show any apparent clinically relevant impact on immune responses to 4CMenB and to the concomitantly administered routine vaccines (DTPa-HBV-IPV/Hib and PCV7) when paracetamol was administered prophylactically to prevent post-immunization fever in children.^12^ The different outcomes might be related to differences in the study design, including vaccination schedule, age of children at the time of vaccination, route of administration of the antipyretic, and different vaccines used for immunization.

The diverging effects of prophylactic paracetamol and ibuprofen administration on vaccine immunogenicity could be explained by differences in the antipyretics mode of action and pharmacokinetics in infants and children.^17^ Ibuprofen non-selectively inhibits both cyclooxygenase (COX)-1 and COX-2, while paracetamol is thought to selectively block COX-3 in brain and spinal cord,^18^ although this latter mechanism of action has been disputed.^19^ While ibuprofen and other non-steroidal anti-inflammatory drugs inhibit cyclooxygenase through competing with arachidonic acid for the active site of the enzyme, paracetamol acts by reducing ferryl protoporphyrin IX at the peroxidase site of the cyclooxygenase enzyme.^18^ Furthermore, it was hypothesized that non-steroidal anti-inflammatory drugs lead to lower levels of produced antibodies due to a decreased expression of B lymphocyte-induced maturation protein 1, which in turn leads to less terminal differentiation of proliferating B-cells into plasma cells.^20^ Unlike ibuprofen, paracetamol inhibits myeloperoxidase-catalyzed oxidant production and, by decreasing hypochlorite production at the inflammation site, could impair immunogenicity by decreasing antigen processing and cross-priming.^21^ Both ibuprofen and paracetamol can rapidly cross the blood-brain barrier, and the latter may act in a synergistic manner on the opioidergic and serotonergic systems.^22,23^

The specific impact of paracetamol on vaccine response could also be explained by the generation of an active metabolite which inhibits the uptake of anandamine and increases its concentration in the brain and blood.^24,25^ Anandamine is a powerful modulator of immune cell functions, especially of primary T cells.^26^ Paracetamol also decreases protein kinase C epsilon translocation in cultured sensory neurons, leading to a decrease of monocyte and macrophage function, as well as Th1 responses.^27,28^ Although no clinical confirmation was found, it has been previously proposed that the impact of paracetamol administration on immune responses to primary vaccination with PHiD-CV may be due to interference with early interactions between dendritic, B and T-cells.^9^ Because this impact is not as evident post-booster, it has been suggested that paracetamol has a higher effect on plasma-cell differentiation than memory-cell differentiation of B-cells. Regardless of the mechanism of action, the overall effect of paracetamol on immunogenicity probably depends on multiple target sites.

For incidence of fever after primary vaccination, results from the confirmatory analysis showed no impact of prophylactic immediate or delayed use of ibuprofen after vaccination. Point estimates of differences in febrile reaction reporting rates between ibuprofen and no-ibuprofen groups during the primary series were close to 0 (immediate manner, reporting rate around 60%) or 10% (delayed manner, reporting rates 61.3 and 51.3%, respectively). Post-booster, no differences in reporting rates of fever between prophylactic ibuprofen groups and the no-ibuprofen group were observed. Yet, immediate or delayed paracetamol administration tended to decrease fever incidence (32.9% and 38.0% of participants, respectively versus 54.1% of participants from the NPARA group).

In another study, prophylactic paracetamol administration effectively prevented fever and other reactions in children vaccinated with PCV7 co-administered with hexavalent vaccine, mainly during the infant series; however, less impact in fever prevention was observed after the booster dose.^29^ This might be explained by an over-estimate of the fever rate after primary vaccination or by a weak anti-inflammatory effect of paracetamol on the more frequent local reactions after booster dose.^29^ Another article reported that the administration of ibuprofen did not induce differences in fever incidence after DTwP or DTaP vaccination, when compared to placebo administration.^30^ Generally, ibuprofen is known to be an antipyretic at least as efficacious as paracetamol.^31-33^ However, limited data are available regarding its prophylactic administration.^11^

Of note, ibuprofen and paracetamol use and labels in the assessed age group differ across countries, which complicated the study set-up and the choice of country in which to perform it. Moreover, ibuprofen is only licensed for use in children from the age of 3 months in Romania or even from 6 months in other European countries, while the first dose of PHiD-CV can be given as early as from the age of 6 weeks. The choice of antipyretic use during pediatric immunization is therefore expected to vary from one country to another, regardless of how the nature of the prophylactic drug impacts the immune response elicited by vaccination.

The study had several strengths: the factorial design addressed all possible combinations of ibuprofen use, a parallel assessment of paracetamol in the same study was performed, and good compliance with the complex study procedures was observed.

A limitation of the current study is that very few results were available from the opsonophagocytic activity and poliomyelitis neutralization assay due to insufficient sera volumes; thus, these results could not be interpreted. In addition, no adjustment for multiplicity was performed for the exploratory group comparisons so the results based on these analyses should be interpreted with caution.

Because prophylactic administration of paracetamol at primary vaccination tends to impact post-primary and post-booster antibody GMCs while ibuprofen was shown not to affect immunogenicity, ibuprofen could be considered as the antipyretic of choice for prophylaxis during primary vaccination courses. However, ibuprofen prophylaxis appeared to have no or only limited effect on fever rates. Thus, prophylactic use of ibuprofen and its benefit/risk ratio should be cautiously considered when deciding in choice of prophylactic antipyretic.

Paracetamol may be more suitable for prevention of febrile reactions after booster vaccination in the second year of life, as it appeared to have no detrimental effect on immunogenicity when administered at booster dose only. However, its use around primary vaccination and benefit/risk ratio should be assessed individually.

Finally, a more conservative approach would be to not provide prophylaxis at all, except when the individual patient would require it. Results of our study may help in guiding general practitioners, pediatricians, and policy makers in their recommendation and choice of antipyretics for prophylaxis of post-vaccination febrile reactions in children.

## Methodology

### Study design and participants

**Figure 3.**
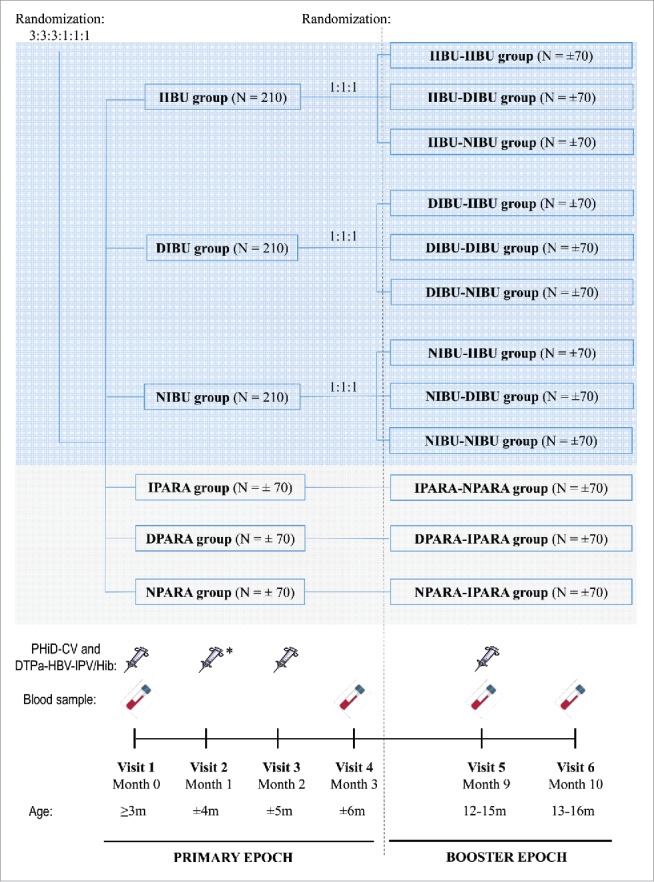
Study design. Footnote: **Primary vaccination**: PHiD-CV and DTPa-(HBV)-IPV/Hib at 3, 4, and 5 months of age, with the following prophylactic antipyretic regimen: IIBU, immediate ibuprofen; DIBU, delayed ibuprofen; NIBU, no ibuprofen; IPARA, immediate paracetamol; DPARA, delayed paracetamol; NPARA, no paracetamol. **Booster vaccination**: PHiD-CV and DTPa-HBV-IPV/Hib at 12–15 months of age, with the following prophylactic antipyretic regimen: at primary vaccination: immediate ibuprofen, and at booster: immediate (IIBU-IIBU), delayed (IIBU-DIBU) or no ibuprofen (IIBU-NIBU); at primary vaccination: delayed ibuprofen, and at booster: immediate (DIBU-IIBU), delayed (DIBU-DIBU) or no ibuprofen (DIBU-NIBU); at primary vaccination: no ibuprofen, and at booster: immediate (NIBU-IIBU), delayed (NIBU-DIBU) or no ibuprofen (NIBU-NIBU); immediate paracetamol at primary vaccination and no paracetamol at booster (IPARA-NPARA); delayed paracetamol at primary vaccination and immediate paracetamol at booster (DPARA-IPARA); no paracetamol at primary vaccination, and immediate paracetamol at booster (NPARA-IPARA). N, number of children per group; m, months; * DTPa-IPV/Hib instead of DTPa-HBV-IPV/Hib.

In this phase IV, multicenter, open-label, randomized, controlled study performed in Romania, infants aged 12–16 weeks at the time of first vaccination (Fig. 3), born after a gestation period of 36–42 weeks and without any obvious health problems, were enrolled. Written informed consent was obtained from each participant's parents or legally authorized representatives. Exclusion criteria are presented in the supplementary material.

The study was conducted according to Good Clinical Practice, the Declaration of Helsinki, and the local rules and regulations of the country; when deviations from these guidelines and regulations were detected, corrective actions were implemented where needed, including exclusion of participants from analyses. This was the case for one study site, at which all study-related activities were terminated during the study due to lack of confidence in the integrity of the data. The infants enrolled at this site were withdrawn from the study, offered continuation of vaccination outside the study and excluded from analyses. As these participants were equally distributed over the different groups, this exclusion had no major impact on the interpretation of the data. The study was registered at www.clinicaltrials.gov (NCT01235949). A protocol summary is available at www.gsk-clinicalstudyregister.com (study ID: 112921).

### Randomization and masking

Enrolled infants were randomized using a blocking scheme (3:3:3:1:1:1) into 3 ibuprofen (IBU) groups and 3 paracetamol (PARA) groups, to receive after each dose of primary vaccinations immediate, delayed, or no ibuprofen or paracetamol prophylactic administration. At booster vaccination, each IBU group (immediate, delayed, or no ibuprofen at priming) was further randomized (1:1:1) into 3 groups (immediate, delayed, or no ibuprofen at booster), while for the 3 PARA groups, treatment (immediate, delayed, or no paracetamol at priming) was re-allocated as defined in the protocol (Fig. 3). The randomization lists were generated at GSK using MATEX for SAS to number the study vaccines and the antipyretic doses given at primary and booster vaccination. Treatment allocation at the site was performed using GSK's internet randomization system (SBIR): the site investigator accessed the randomization system on the internet and provided the identification number for eligible infants. The randomization system then used a minimization algorithm to determine the treatment number for the study vaccines and antipyretic doses to be used for the infant.

The study was conducted in an open manner; the participants' parent(s) or legally acceptable representative, the investigator, and all study staff involved in the clinical evaluation of participants were aware of treatment allocation.

### Procedures

Participants received 3-dose primary vaccination with PHiD-CV (*Synflorix*™, GSK, Belgium) at 3, 4, and 5 months of age and booster dose at 12–15 months of age (intramuscular, in the right thigh, or deltoid for children >12 months); 2 doses of DTPa-HBV-IPV/Hib (*Infanrix hexa*™, GSK, Belgium) at 3 and 5 months of age and booster dose at 12–15 months of age (intramuscular, left thigh or deltoid); and one dose of DTPa-IPV/Hib (*Infanrix-IPV/Hib*™, GSK, Belgium) at 4 months of age (intramuscular, left thigh). The first dose of antipyretic (ibuprofen (*Nurofen*™, Reckitt Benckiser, UK) – 10 mg/kg/dose, with a maximum daily dose of 30 mg/kg, or paracetamol (*Panadol Baby*™, GSK, UK) – 15 mg/kg/dose with a maximum daily dose of 60 mg/kg) was administered orally either immediately after vaccination at the study site (immediate administration) or by the parents at home 4–6 hours after vaccination (delayed administration). The second and third dose of antipyretic were administered by the parents at home, 6–8 hours after the previous dose; if a child slept overnight, the dose was deferred to the following morning.

### Outcomes

The primary study outcome was to assess the percentage of infants with anti-pneumococcal antibody concentrations ≥0.2 µg/mL for each of the 10 PHiD-CV serotypes, in order to demonstrate non-inferiority of immune response to PHiD-CV administered as a 3-dose primary vaccination course with immediate or delayed prophylactic ibuprofen compared to PHiD-CV without prophylactic ibuprofen administration.

Secondary outcomes included determination of the percentage reduction in fever episodes with immediate or delayed prophylactic ibuprofen administration after primary PHiD-CV vaccination (confirmatory objective). The percentage of participants with local and general adverse events within 4 days, with unsolicited AEs within 31 d after each vaccine dose, and the occurrence of SAEs during the entire study were also assessed. Another secondary outcome was the evaluation of the immune responses to the components of PHiD-CV and the co-administered DTPa-HBV-IPV/Hib and DTPa-IPV/Hib vaccines, in terms of antibody concentrations one month post-primary immunization, prior to and one month after booster immunization.

## Statistical analysis

### Immunogenicity analysis

Immunogenicity analyses were performed for the primary and booster ATP immunogenicity cohort, comprising all evaluable participants (meeting all eligibility criteria and no elimination criteria, who complied with protocol-defined procedures/intervals) with results available for primary or booster immunogenicity endpoint measures.

### Confirmatory inferential analysis for the primary objective

The global type I error for each pair-wise comparison was adjusted to 1.25% using a Bonferroni adjustment to ensure that the overall type I error was below 2.5%, considering that the 2 IBU groups were compared to the control group (without ibuprofen). The non-inferiority to the control group was further adjusted to account for endpoint multiplicity using the method by Lehman et al.,^34^ leading to a nominal type I error = 1.25%*(7/10) = 0.875%. The statistical decrease in GMC was also adjusted to account for the 11 endpoints (10 serotypes and anti-protein D) using a Bonferroni adjustment, leading to a nominal type I error = 1.25%/11 = 0.11364%.

The study had no less than 92.1% power to detect a statistical difference for a true GMC decrease equal to 2-fold. To obtain a power of 92.7% using an adjusted one-sided α of 0.875%, a sample size of 180 participants for each primary IBU group was necessary. Anticipating that ∼14% of vaccinated participants would not be evaluable for the ATP cohort for immunogenicity, we planned to enroll 210 participants in each ibuprofen group.

Standardized asymptotic 98.25% CIs were computed using StatXact for the difference between groups in the percentage of participants with anti-pneumococcal antibody concentrations ≥ 0.2 µg/mL one month post-dose 3 (NIBU minus IIBU, or NIBU minus DIBU). Non-inferiority was demonstrated for one of the 2 pair-wise group comparisons if the UL of the 2-sided 98.25% CI was below 10% for at least 7 of the 10 vaccine pneumococcal serotypes.

99.8% CIs for antibody GMC ratios (IIBU/NIBU and DIBU/NIBU), one month post-dose 3, were computed for each of the 10 vaccine pneumococcal serotypes and protein D using a one-sided ANOVA test on the logarithm10 transformation of the concentrations. A statistically significant difference in post-dose 3 antibody GMCs was established if the UL of the 2-sided 99.8% CI was <1 for at least one of the 10 vaccine pneumococcal serotypes or protein D.

### Factorial analysis

The nine randomized booster IBU groups were designed to enable a factorial analysis in which 2 factors (factor A – ibuprofen administration at primary vaccination, factor B – ibuprofen administration at booster vaccination), and 3 levels for each factor (immediate, delayed or no ibuprofen administration), could be evaluated. Further details are provided in supplementary methods.

### Exploratory analyses

The exploratory analyses for the IBU and PARA groups are detailed in the supplementary methods. Briefly, the exclusion of 0 from the 95% CI of difference between groups in percentage of participants with antibody concentrations above the threshold, and the exclusion of 1 from the 95% CI of antibody GMC ratios were used to highlight potential group differences. For other comparisons, non-overlapping 95% CIs were used as indicator of potential differences.

### Within-group assessments

Antibody GMCs with 95% CIs were tabulated for each group, at each timepoint with a blood sample result available, and seropositivity/seroprotection rates with exact 95% CIs were calculated for each appropriate serotype/antigen. Antibody GMC calculations were performed by taking the anti-log of the mean of the log concentration transformations. Antibody concentrations below the cut-off of the assay were given an arbitrary value of half the cut-off for the purpose of GMC calculation. The 95% CI for the mean of log-transformed concentration was first obtained, assuming that log-transformed values were normally distributed with unknown variance. The 95% CI for the GMCs was then obtained by exponential-transformation of the 95% CI for the mean of the log-transformed concentration.

### Safety analysis

Safety analyses were performed for the TVC, comprising all children who received at least one primary vaccine dose (primary TVC) or the booster dose (booster TVC). Because more than 5% of vaccinated participants were excluded from the ATP safety cohort, a complementary analysis was performed based on this ATP cohort, which included participants who met all eligibility criteria and with no elimination criteria, who had received at least one vaccine dose and antipyretic (if applicable) according to their random assignment (primary ATP cohort for safety analysis) or who had received all primary vaccine doses with antipyretic (if applicable) plus the booster dose and antipyretic, if applicable (booster ATP cohort for safety analysis), in compliance with the protocol-defined vaccine administration route and antipyretic dose.

### Confirmatory inferential analysis

Standardized asymptotic 97.5% CIs for the difference between groups in percentage of participants with rectal temperature ≥38°C within 4 d after at least one primary dose (NIBU minus IIBU, or NIBU minus DIBU) were computed using StatXact. This secondary confirmatory objective was assessable if the primary objective was reached, and was demonstrated if the lower limit (LL) of the 97.5% CI around the difference was higher than 0%.

## Supplementary Material

Supplementary Materials
