# Supplementary material for: Effects of prophylactic ibuprofen and paracetamol administration on the immunogenicity and reactogenicity of the 10-valent pneumococcal non-typeable Haemophilus influenzae protein D conjugated vaccine (PHiD-CV) co-administered with DTPa-combined vaccines in children: An open-label, randomized, controlled, non-inferiority trial
Source: Hum Vaccin Immunother. 2016 Aug 19;13(3):649–60. doi: 10.1080/21645515.2016.1223001 (PMC5360152; doi:10.1080/21645515.2016.1223001)
Supplement: Supplementary Materials [file khvi-13-03-1223001-s001.pdf]

Supplementary material

Exploratory inferential analysis

95% CIs for the antibody GMC ratios (GMCs from the IIBU group over the GMCs from NIBU group, and GMCs from the DIBU group over the GMCs from NIBU group) one month post-dose three, were computed for each of the antigens included in DTPa-HBV-IPV/Hib vaccine. 95% CIs for the antibody GMC ratios (GMCs from the IPARA group over the GMCs from NPARA group, and GMCs from the DPARA group over the GMCs from NPARA group) one month post-dose three, were computed for each of the 10 vaccine pneumococcal serotypes, for protein D and for each of the antigens included in DTPa-HBV-IPV/Hib vaccine. Standardized asymptotic 95% CIs for the difference between groups (IPARA group minus NPARA group and DPARA group minus NPARA group), in percentage of participants with anti-pneumococcal antibody concentrations ≥ 0.2 μg/mL one month post-dose three, were computed.

Factorial design analysis

The nine booster groups, organized in a factorial design, were compared in terms of antibody GMCs for each of the ten vaccine pneumococcal serotypes, for protein D and for each of the antigens included in DTPa-HBV-IPV/Hib vaccine using a two-way ANOVA F-test (ANOVA model including Factor A, Factor B, and the interaction between A and B). If a statistically significant difference was found (p-value < 0.025), pair-wise comparisons were made using Tukey's multiple comparison adjustment.

The antibody GMC ratio between two groups and its confidence interval (99.8% CI for primary objective, 95% CI for exploratory analyses) were obtained using an ANOVA model on the logarithm-transformed concentrations. The ANOVA model only included the vaccine group as fixed effect. The antibody GMC ratio and its 95% CI were derived as exponential-transformation of the corresponding group contrast in the model. Proc StatXact 8.1 or Proc Genmod from SAS 9.22 was used to derive the standardized asymptotic CI (98.25% CI for immunogenicity, 97.5% CI for reactogenicity) for the group difference in proportions using method 6 as described by R.G. Newcombe.[1]

Based on the factorial design and considering that around 540 participants would enter the booster phase, of which 10% would not be evaluable for immunogenicity analysis one month post-booster, 486 evaluable participants were planned to be enrolled (54 participants per booster group).

Exclusion criteria – Supplementary Information

Participants were excluded from the study if any of the following occurred:

- Administration within 30 days preceding the first dose of study vaccine/product, or planned use during the study period of any of the following: drug or vaccine other than the study vaccines/products; chronic administration of immunosuppressants (for corticosteroids, this meant prednisone, or equivalent, ≥ 0·5 mg/kg/day. Inhaled and topical steroids were allowed); administration of immunoglobulins and/or any blood products. Treatment with antipyretics in the 24 hours before study vaccination or planned administration of antipyretics in the 24 hours after study vaccination. Other indication than specified in the protocol for prophylactic or therapeutic antipyretic treatment during the study period.
- Previous vaccination against diphtheria, tetanus, pertussis, polio, hepatitis B, *H. influenzae* type b and/or *S. pneumoniae* with the exception of the vaccines where the first dose could have been given within the first two weeks of life according to the national recommendations.
- Concurrently participating in another clinical study, at any time during the study period, in which the participant had been or would have been exposed to an investigational or a non-investigational product (pharmaceutical product or device).
- History of intercurrent diphtheria, tetanus, pertussis, polio, hepatitis B, *H. influenzae* type b disease.
- History of allergic disease or reaction likely to be exacerbated by any component of the vaccines or prophylactic antipyretic treatment, i.e. ibuprofen or paracetamol, as specified in the protocol; history of seizures or progressive neurological disease; family history of congenital or hereditary immunodeficiency.
- Acute disease and/or fever (defined as oral, axillary or tympanic temperature ≥37.5°C or rectal temperature ≥38.0°C) at the time of enrolment. Participants with a minor illness (such as mild diarrhea, mild upper respiratory infection) without fever could have been enrolled at the discretion of the investigator.
- Any confirmed or suspected immunosuppressive or immunodeficient condition.
- Major congenital defects or serious chronic illness.
- Any contraindication to treatment with ibuprofen or paracetamol as described in the summary of product characteristics of the antipyretic.

- Body weight < 5 kg at the time of enrolment (For participants with a body weight < 5 kg at the time of first vaccination, the first vaccination visit warranted deferral to comply with the body weight criterion for antipyretics administration).
- Child in care.

Secondary objectives – Supplementary Information

- To determine the percentage reduction in febrile reactions (rectal temperature ≥38·0°C) when immediate or delayed ibuprofen is administered compared to no prophylactic ibuprofen administration, after primary vaccination with PHiD-CV co-administered with DTPa-combined vaccines.
- To assess the impact of immediate or delayed prophylactic paracetamol administration on PHiD-CV immunogenicity and on the incidence of febrile reactions after PHiD-CV vaccination co-administered with DTPa-combined vaccines, after primary vaccination and after booster vaccination.
- To assess the impact of immediate or delayed paracetamol administration on the incidence of febrile reactions after PHiD-CV booster vaccination co-administered with DTPa-combined vaccines.
- To assess, prior to booster vaccination, the impact of immediate or delayed ibuprofen or paracetamol administration on the persistence of antibodies induced by PHiD-CV and DTPa-combined vaccines given as a primary vaccination course.
- To assess the impact of immediate or delayed ibuprofen or paracetamol administration on the immunogenicity of a booster dose of PHiD-CV co-administered with DTPa-combined vaccine.

Study procedures – Supplementary information

All study vaccines were manufactured by GSK Vaccines.

PHiD-CV vaccine is a 10-valent pneumococcal vaccine, each dose (0.5mL) containing: 1 µg of each capsular polysaccharide for serotypes 1, 5, 6B, 7F, 9V, 14, and 23F and 3 µg for serotype 4, individually conjugated to protein D; 3 µg of capsular polysaccharide of serotype 18C conjugated to conjugated to tetanus toxoid; and 3 µg of capsular polysaccharide of serotype 19F conjugated to diphtheria toxoid.

One dose (0.5mL) of DTPa-HBV-IPV/Hib vaccine (contains at least 30 IU diphtheria toxoid; at least 40 IU tetanus toxoid; 25 µg pertussis toxin; 25 µg filamentous haemagglutinin; 8 µg pertactin; 10 µg recombinant hepatitis

B surface antigen (HBsAg); 40D, 8D, and 32D antigen units of poliovirus types 1, 2, and 3, respectively; and 10 µg *H. influenzae* type b polyribosyl-ribitol- phosphate conjugated to tetanus toxoid.

One dose (0.5mL) of DTPa-IPV/Hib vaccine (contains at least 30 IU diphtheria toxoid; at least 40 IU tetanus toxoid; 25 µg pertussis toxin; 25 µg filamentous haemagglutinin; 8 µg pertactin; 40D, 8D, and 32D antigen units of poliovirus types 1, 2, and 3, respectively; and 10 µg *H. influenzae* type b polyribosyl-ribitol- phosphate conjugated to tetanus toxoid.

Immunogenicity assessment

Blood samples were collected pre- and one month post-primary vaccination, and pre- and one month post-booster. Concentrations of antibodies against vaccine antigens were measured by enzyme-linked immunosorbent assay (22F-ELISA): vaccine and vaccine-related pneumococcal serotypes 6A and 19A, anti-diphtheria, anti-tetanus and anti-polyribosyl-ribitol phosphate [anti-PRP], anti-pertussis components (pertussis toxoid [PT], filamentous haemagglutinin [FHA] and pertactin [PRN]), and anti-protein D. Antibodies against hepatitis B surface antigen (HBs) were determined using Centaur® and ChemiLuminescence ImmunoAssay (CLIA) assay. For the measurement of the serotype-specific pneumococcal antibodies, the 22F-inhibition ELISA was used; detailed description of the assay is available elsewhere.[2, 3] A threshold of 0.2 µg/mL anti-pneumococcal antibody concentrations was used to demonstrate the primary objective (this level is equivalent to the antibody concentration of 0.35 µg/mL measured by the non-22F ELISA of the WHO reference laboratory).[2] Seropositivity/seroprotection rates with exact 95% CIs were calculated for each appropriate serotype/antigen. Seropositivity was defined as ≥5 ELISA units (EL.U)/mL for anti-PT, anti-FHA, and anti-PRN antibodies, and ≥100 EL.U/mL for anti-protein D antibodies. Seroprotection was defined as anti-diphtheria and anti-tetanus antibody concentrations ≥0.1 IU/mL, anti-PRP antibody concentrations ≥0.15 µg/mL, and anti-HBs antibody concentrations ≥10 mIU/mL.

Safety assessment

Solicited local (pain, redness or swelling at the injection site) or general (drowsiness, fever, irritability/fussiness or loss of appetite) adverse events (AEs) were recorded during four days post-vaccination on the participant’s diary card. Unsolicited AEs were recorded during 31 days post-vaccination and serious AEs (SAEs) throughout the study.

Safety results – Supplementary information

During the primary epoch, the maximum overall/dose incidence for grade 3 solicited local and general symptoms in each group was 4.2% and 2.4%, respectively. Post-booster, grade 3 solicited local and general symptoms were reported by maximum 7.9% and 5.1% of participants, respectively (Supplementary figures 1, 2). Grade 3 solicited general symptoms assessed by the investigator to be causally related to vaccination were reported after maximum 1.7% of primary doses, and by maximum 5.1% of participants post-booster.

During the primary phase, unsolicited symptoms were reported following maximum 9.5% of doses per group. Grade 3 unsolicited symptoms were reported after 1 dose (0.2%) in the DIBU group (bronchiolitis), after 1 dose (0.2%) in the NIBU group (bronchiolitis), and after 1 dose (0.5%) in the IPARA group (acute respiratory failure). An unsolicited symptom assessed by the investigator to have a causal relationship with vaccination was reported after 2 doses (0.3%) in the IIBU group (injection site nodule), after 3 doses (0.5%) in the DIBU group (vomiting) and after 2 doses (1.0%) in the IPARA group (1 case of diarrhea and 1 case of soft feces); none of these related symptoms were of grade 3 severity.

Post-booster, unsolicited symptoms were reported for maximum 10.0% of participants per group. An unsolicited symptom with causal relationship to vaccination (nodule) was reported for one participant (1.6%) in the IIBU-IIBU group; this symptom was of grade 3 severity. Another grade 3 unsolicited symptom (convulsion), which was not considered to be causally related to vaccination, was reported for one participant (1.5%) in the IPARA-NPARA group.

Factorial design analysis results – Supplementary information

For the co-administered vaccines, only ibuprofen administration at the time of primary vaccination (factor A) was found to have a borderline significant effect on post-booster anti-tetanus responses (Supplementary table 6). Because no statistically significant interaction was observed using a two-way ANOVA F-test, a two-way ANOVA without interaction was run. Again, only factor A for the tetanus antigen was found to have a statistically significant impact ($p = 0.0396$). The subsequent Tukey's multiple comparison adjustment test showed a statistically significant difference for IIBU vs NIBU (GMC ratio: 0.78 [adjusted 95% CI: 0.62; 0.99], borderline significance).

1. Newcombe RG. Interval estimation for the difference between independent proportions: comparison of eleven methods. Statistics in medicine 1998; 17: 873-90.
2. Henckaerts I, Goldblatt D, Ashton L, Poolman J. Critical differences between pneumococcal polysaccharide enzyme-linked immunosorbent assays with and without 22F inhibition at low antibody concentrations in pediatric sera. Clinical and vaccine immunology 2006; 13:356-60.
3. Poolman JT, Frasch CE, Kayhty H et al. Evaluation of pneumococcal polysaccharide immunoassays using a 22F adsorption step with serum samples from infants vaccinated with conjugate vaccines. Clinical and Vaccine Immunology 2010; 17:134-42.

Supplementary figure 1. Solicited symptoms on days 0-3 post-primary dose

A. Injection site pain

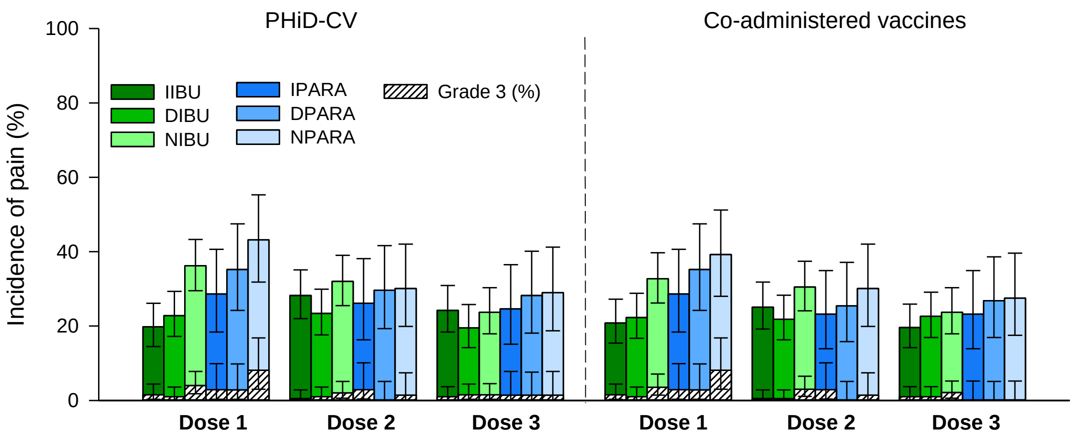

B. Injection site redness

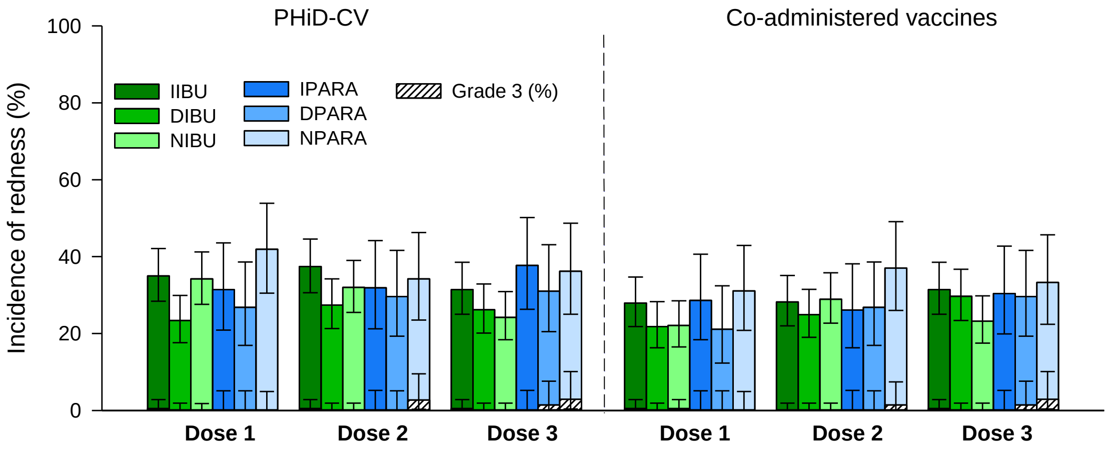

C. Injection site swelling

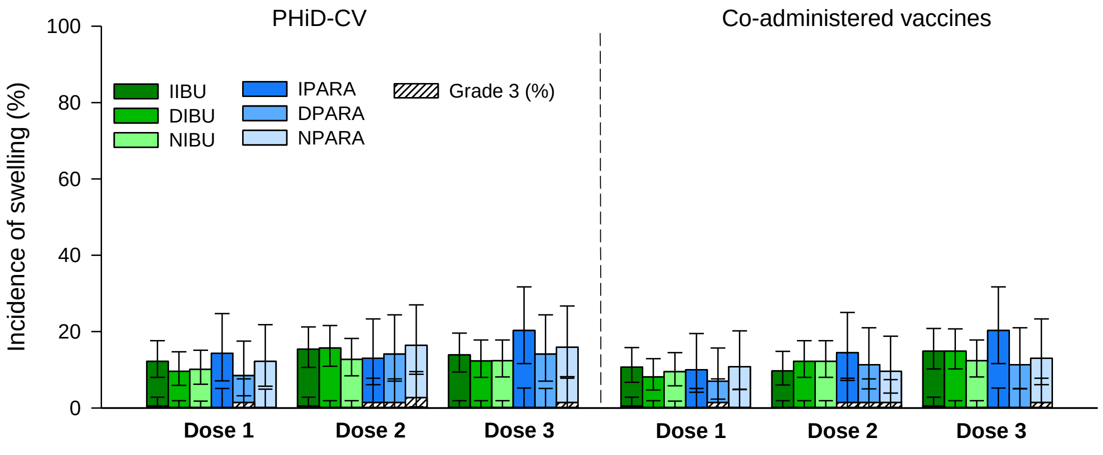

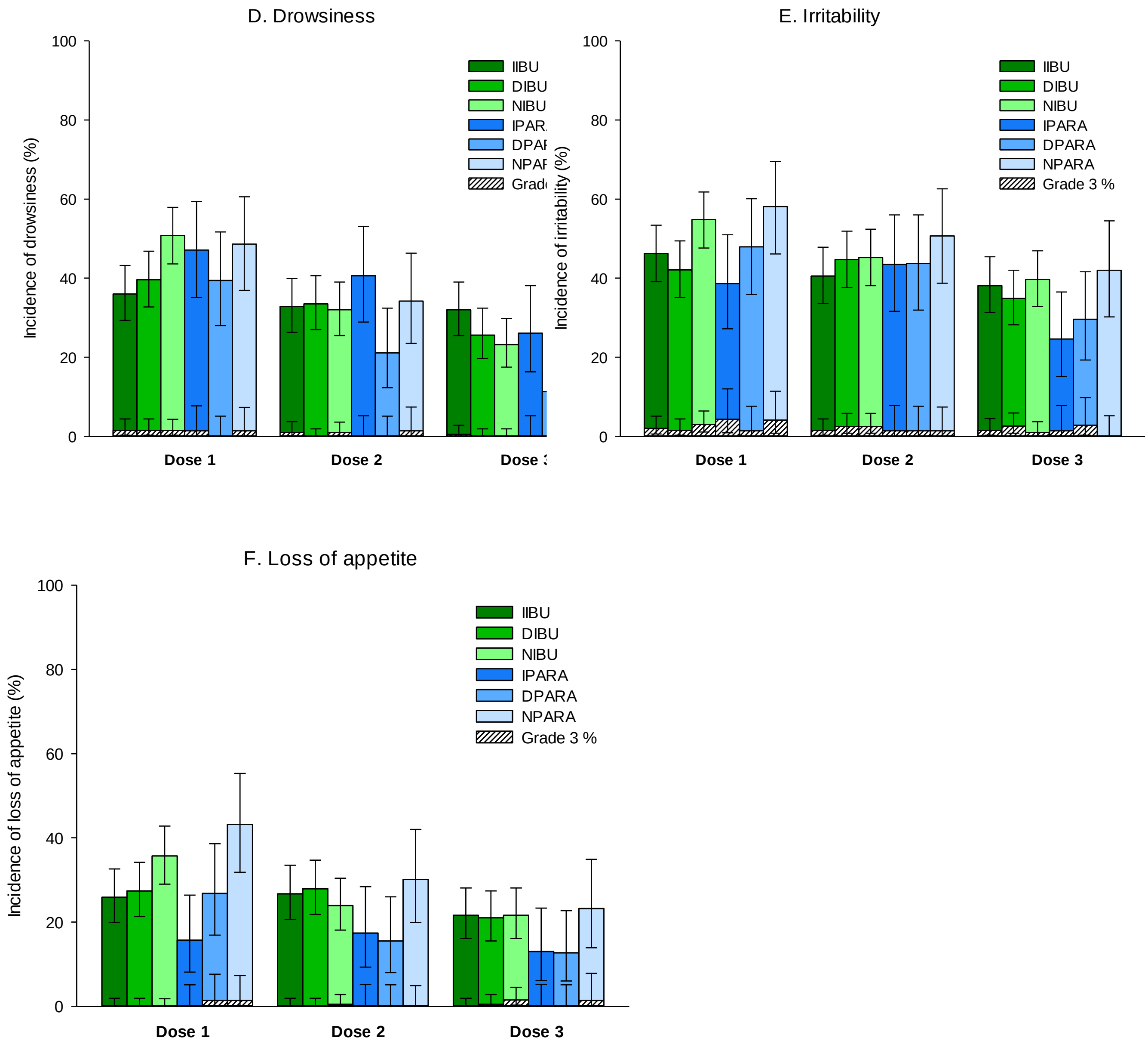

Footnote: PHiD-CV and DTPa-(HBV)-IPV/Hib at 3, 4, and 5 months of age, with the following prophylactic antipyretic regimen: IIBU, immediate ibuprofen; DIBU, delayed ibuprofen; NIBU, no ibuprofen; IPARA, immediate paracetamol; DPARA, delayed paracetamol; NPARA, no paracetamol; %, percentage of children.

Grade 3 was reported for pain at the injection site if the child cried when limb was moved/spontaneously painful; for injection site redness /swelling >30 mm; for drowsiness if normal activity was prevented; for irritability/fussiness if child cried and could not be comforted or if normal activity was prevented; for loss of appetite if the child was not eating at all.

Supplementary figure 2. Solicited symptoms on days 0-3 post-booster dose

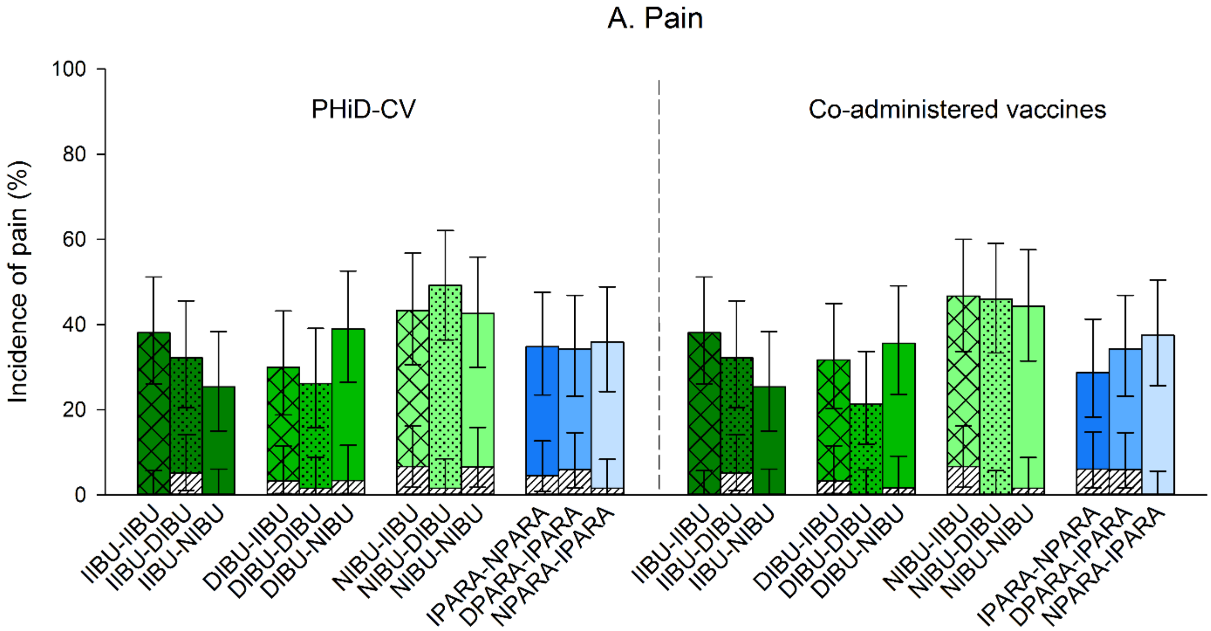

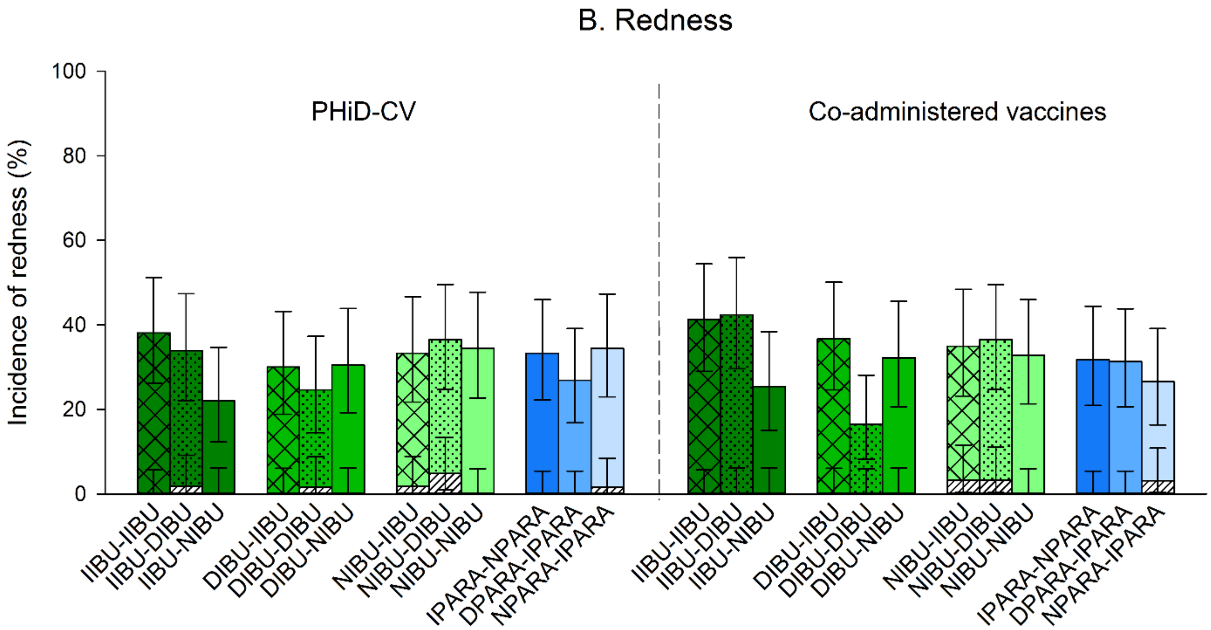

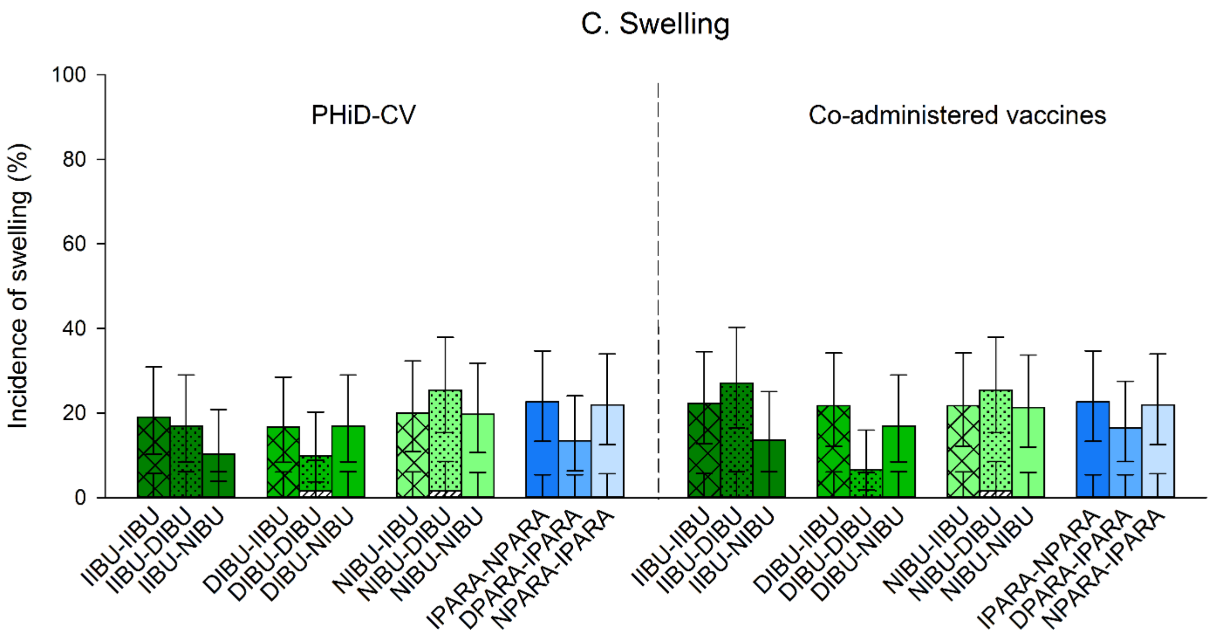
C. Swelling
PHiD-CV
Co-administered vaccines
Incidence of swelling (%)
100
80
60
40
20
0
IIBU-IIBU
IIBU-DIBU
IIBU-NIBU
DIBU-IIBU
DIBU-DIBU
DIBU-NIBU
NIBU-IIBU
NIBU-DIBU
NIBU-NIBU
IPARA-NPARA
DPARA-IPARA
NPARA-IPARA

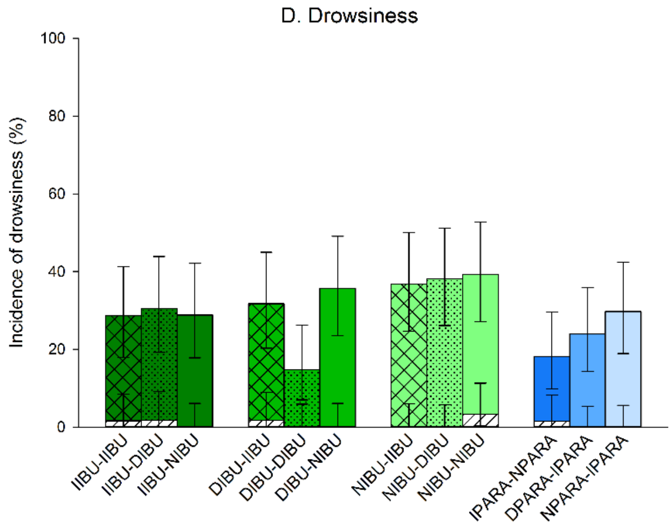
D. Drowsiness
Incidence of drowsiness (%)
100
80
60
40
20
0
IIBU-IIBU
IIBU-DIBU
IIBU-NIBU
DIBU-IIBU
DIBU-DIBU
DIBU-NIBU
NIBU-IIBU
NIBU-DIBU
NIBU-NIBU
IPARA-NPARA
DPARA-IPARA
NPARA-IPARA

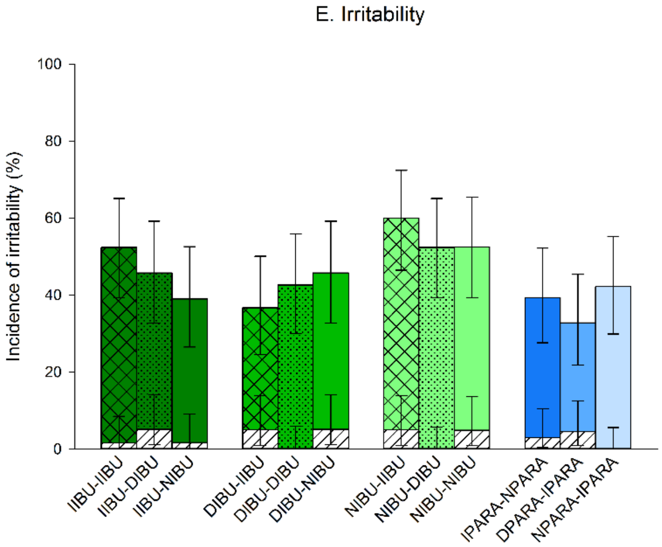
E. Irritability
Incidence of irritability (%)
100
80
60
40
20
0
IIBU-IIBU
IIBU-DIBU
IIBU-NIBU
DIBU-IIBU
DIBU-DIBU
DIBU-NIBU
NIBU-IIBU
NIBU-DIBU
NIBU-NIBU
IPARA-NPARA
DPARA-IPARA
NPARA-IPARA

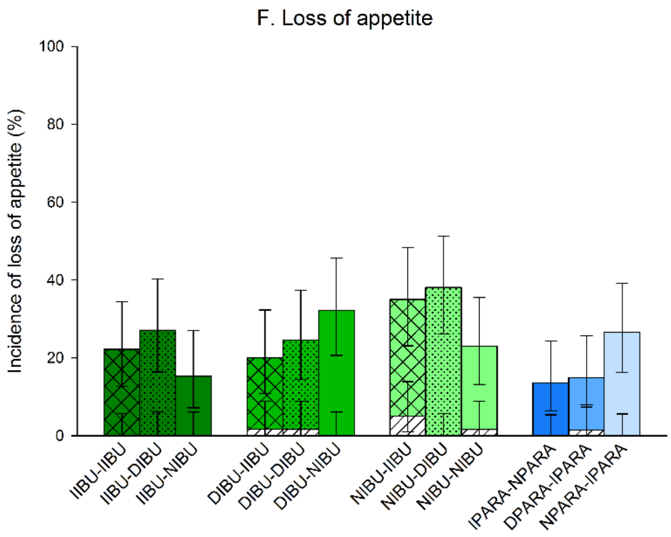

Footnote: PHiD-CV and DTPa-HBV-IPV/Hib at 12-15 months of age, with the following prophylactic antipyretic regimen: at primary vaccination: immediate ibuprofen, and at booster: immediate (IIBU-IIBU), delayed (IIBU-DIBU) or no ibuprofen (IIBU-NIBU); at primary vaccination: delayed ibuprofen, and at booster: immediate (DIBU-IIBU), delayed (DIBU-DIBU) or no ibuprofen (DIBU-NIBU); at primary vaccination: no ibuprofen, and at booster: immediate (NIBU-IIBU), delayed (NIBU-DIBU) or no ibuprofen (NIBU-NIBU); immediate paracetamol at primary vaccination and no paracetamol at booster (IPARA-NPARA); delayed paracetamol at primary vaccination and immediate paracetamol at booster (DPARA-IPARA); No paracetamol at primary vaccination, and immediate paracetamol at booster (NPARA-IPARA); %, percentage of children.
Grade 3 was reported for pain at the injection site if the child cried when limb was moved/spontaneously painful; for injection site redness /swelling >30 mm; for drowsiness if normal activity was prevented; for irritability/fussiness if child cried and could not be comforted or if normal activity was prevented; for loss of appetite if the child was not eating at all.

Supplementary table 1. Demographic characteristics (total vaccinated cohort)

Primary epoch

**Characteristics**	**IIBU N = 198**	**DIBU N = 198**	**NIBU N = 199**	**IPARA N = 71**	**DPARA N = 72**	**NPARA N = 74**	**Total N = 812**
Mean age (weeks) at vaccine dose 1, SD	13.1 (1.15)	13.0 (1.20)	12.9 (1.14)	13.0 (1.30)	13.2 (1.30)	13.2 (1.11)	13.1 (1.18)
Mean age (weeks) at vaccine dose 2, SD	18.1 (1.46)	18.0 (1.56)	17.9 (1.36)	18.0 (1.64)	18.2 (1.51)	18.1 (1.49)	18.0 (1.48)
Mean age (weeks) at vaccine dose 3, SD	23.1 (1.72)	23.2 (1.85)	23.0 (1.76)	22.8 (1.78)	23.3 (1.73)	23.2 (1.82)	23.1 (1.78)
Male (n, %)	108 (54.5)	112 (56.6)	95 (47.7)	32 (45.1)	32 (44.4)	36 (48.6)	415 (51.1)
White - Caucasian / European Heritage (n, %)	198 (100)	197 (99.5)	198 (99.5)	70 (98.6)	72 (100)	72 (97.3)	807 (99.4)
Total dose (mg/kg) of administered prophylactic antipyretic following vaccine dose 1, mean	27.73	27.79	-	41.88	42.08	-	-
Total dose (mg/kg) of administered prophylactic antipyretic following vaccine dose 2, mean	26.60	26.57	-	41.61	41.34	-	-
Total dose (mg/kg) of administered prophylactic antipyretic following vaccine dose 3, mean	26.16	26.51	-	41.13	41.56	-	-

Booster epoch

**Characteristics**	**IIBU-IIBU N = 64**	**IIBU-DIBU N = 60**	**IIBU-NIBU N = 63**	**DIBU-IIBU N = 63**	**DIBU-DIBU N = 63**	**DIBU-NIBU N = 63**	**NIBU-IIBU N = 63**	**NIBU-DIBU N = 65**	**NIBU-NIBU N = 62**	**IPARA-NPARA N = 67**	**DPARA-IPARA N = 68**	**NPARA-IPARA N = 67**	**Total N = 768**
Mean age (months) at booster dose, SD	12.3 (0.64)	12.2 (0.49)	12.3 (0.57)	12.2 (0.45)	12.4 (0.63)	12.4 (0.73)	12.3 (0.63)	12.3 (0.57)	12.3 (0.70)	12.3 (0.73)	12.3 (0.56)	12.4 (0.63)	12.3 (0.62)
Male (n, %)	35 (54.7)	33 (55.0)	34 (54.0)	40 (63.5)	30 (47.6)	36 (57.1)	31 (49.2)	30 (46.2)	27 (43.5)	30 (44.8)	29 (42.6)	34 (50.7)	389 (50.7)
White - Caucasian / European Heritage (n, %)	64 (100)	60 (100)	63 (100)	63 (100)	63 (100)	62 (98.4)	63 (100)	65 (100)	62 (100)	66 (98.5)	68 (100)	66 (98.5)	765 (99.6)
Total dose (mg/kg) of administered prophylactic antipyretic after booster dose	26.71	26.53	-	26.62	26.92	-	26.41	26.56	-	-	42.19	41.09	-

Footnote: **Primary vaccination**: PHiD-CV and DTPa-(HBV)-IPV/Hib at 3, 4, and 5 months of age, with the following prophylactic antipyretic regimen: IIBU, immediate ibuprofen; DIBU, delayed ibuprofen; NIBU, no ibuprofen; IPARA, immediate paracetamol; DPARA, delayed paracetamol; NPARA, no paracetamol.

Booster vaccination: PHiD-CV and DTPa-HBV-IPV/Hib at, with the following prophylactic antipyretic regimen: at primary vaccination: immediate ibuprofen, and at booster: immediate (IIBU-IIBU), delayed (IIBU-DIBU) or no ibuprofen (IIBU-NIBU); at primary vaccination: delayed ibuprofen, and at booster: immediate (DIBU-IIBU), delayed (DIBU-DIBU) or no ibuprofen (DIBU-NIBU); at primary vaccination: no ibuprofen, and at booster: immediate (NIBU-IIBU), delayed (NIBU-DIBU) or no ibuprofen (NIBU-NIBU); immediate paracetamol at primary vaccination and no paracetamol at booster (IPARA-NPARA); delayed paracetamol at primary vaccination and immediate paracetamol at booster (DPARA-IPARA); No paracetamol at primary vaccination, and immediate paracetamol at booster (NPARA-IPARA).

N, total number of participants; n/%, number/percentage of participants in a given category; SD, standard deviation.

Supplementary table 2. Pre- and post-booster antibody responses to PHiD-CV for the IBU groups (ATP cohort for immunogenicity)

Serotype		Percentage of children with antibody concentrations ≥ 0.2 µg/mL (95% CI)								
		**IIBU-IIBU N = 50**	**IIBU-DIBU N = 46**	**IIBU-NIBU N = 43**	**DIBU-IIBU N = 48**	**DIBU-DIBU N = 48**	**DIBU-NIBU N = 47**	**NIBU-IIBU N = 47**	**NIBU-DIBU N = 47**	**NIBU-NIBU (Control) N = 46**
Vaccine serotypes:										
**1**	PRE	80.4 (66.1; 90.6)	79.5 (64.7; 90.2)	67.5 (50.9; 81.4)	70.2 (55.1; 82.7)	81.8 (67.3; 91.8)	75.0 (59.7; 86.8)	82.6 (68.6; 92.2)	79.5 (64.7; 90.2)	80.0 (65.4; 90.4)
	POST	100 (92.7; 100)	95.7 (85.2; 99.5)	97.2 (85.5; 99.9)	100 (92.0; 100)	97.9 (88.9; 99.9)	95.7 (85.2; 99.5)	97.7 (88.0; 99.9)	100 (92.1; 100)	97.6 (87.1; 99.9)
**4**	PRE	97.9 (88.7; 99.9)	93.3 (81.7; 98.6)	92.5 (79.6; 98.4)	91.5 (79.6; 97.6)	91.3 (79.2; 97.6)	91.1 (78.8; 97.5)	95.7 (85.2; 99.5)	93.5 (82.1; 98.6)	88.9 (75.9; 96.3)
	POST	100 (92.6; 100)	100 (92.1; 100)	100 (90.3; 100)	97.8 (88.2; 99.9)	100 (92.5; 100)	100 (92.3; 100)	100 (91.6; 100)	100 (92.1; 100)	100 (91.4; 100)
**5**	PRE	100 (92.3; 100)	97.7 (88.0; 99.9)	95.1 (83.5; 99.4)	93.6 (82.5; 98.7)	95.6 (84.9; 99.5)	95.5 (84.5; 99.4)	100 (92.3; 100)	100 (92.1; 100)	100 (92.1; 100)
	POST	100 (92.6; 100)	100 (92.1; 100)	100 (90.3; 100)	100 (92.0; 100)	100 (92.6; 100)	100 (92.3; 100)	100 (91.8; 100)	100 (92.1; 100)	100 (91.4; 100)
**6B**	PRE	87.8 (75.2; 95.4)	84.4 (70.5; 93.5)	87.8 (73.8; 95.9)	85.4 (72.2; 93.9)	91.7 (80.0; 97.7)	89.4 (76.9; 96.5)	87.0 (73.7; 95.1)	91.3 (79.2; 97.6)	95.6 (84.9; 99.5)
	POST	100 (92.7; 100)	97.8 (88.2; 99.9)	100 (90.3; 100)	100 (92.1; 100)	95.8 (85.7; 99.5)	91.5 (79.6; 97.6)	95.5 (84.5; 99.4)	95.7 (85.2; 99.5)	100 (91.6; 100)
**7F**	PRE	100 (92.5; 100)	100 (91.8; 100)	100 (91.4; 100)	93.6 (82.5; 98.7)	100 (92.3; 100)	100 (92.1; 100)	100 (92.3; 100)	100 (92.1; 100)	97.7 (88.0; 99.9)
	POST	100 (92.6; 100)	100 (92.1; 100)	100 (90.3; 100)	100 (92.0; 100)	100 (92.6; 100)	100 (92.5; 100)	100 (91.6; 100)	100 (92.1; 100)	100 (91.4; 100)
**9V**	PRE	97.9 (88.9; 99.9)	97.8 (88.2; 99.9)	100 (91.6; 100)	93.6 (82.5; 98.7)	100 (92.5; 100)	100 (92.3; 100)	97.8 (88.5; 99.9)	97.8 (88.5; 99.9)	100 (92.1; 100)
	POST	100 (92.7; 100)	97.8 (88.5; 99.9)	100 (90.3; 100)	100 (92.1; 100)	100 (92.6; 100)	100 (92.5; 100)	100 (91.8; 100)	100 (92.5; 100)	100 (91.6; 100)
**14**	PRE	95.7 (85.5; 99.5)	100 (92.0; 100)	90.2 (76.9; 97.3)	95.7 (85.5; 99.5)	97.8 (88.5; 99.9)	97.8 (88.5; 99.9)	93.5 (82.1; 98.6)	97.8 (88.5; 99.9)	97.8 (88.2; 99.9)
	POST	100 (92.7; 100)	97.8 (88.5; 99.9)	100 (90.7; 100)	100 (92.1; 100)	100 (92.6; 100)	100 (92.5; 100)	100 (92.0; 100)	100 (92.3; 100)	100 (91.8; 100)
**18C**	PRE	98.0 (89.4; 99.9)	97.7 (88.0; 99.9)	100 (91.4; 100)	95.7 (85.5; 99.5)	100 (92.6; 100)	100 (92.3; 100)	100 (92.3; 100)	97.8 (88.5; 99.9)	95.7 (85.2; 99.5)
	POST	100 (92.6; 100)	100 (92.1; 100)	100 (90.7; 100)	100 (92.0; 100)	100 (92.6; 100)	100 (92.5; 100)	100 (92.1; 100)	97.8 (88.5; 99.9)	100 (91.6; 100)
**19F**	PRE	97.8 (88.5; 99.9)	100 (92.0; 100)	100 (91.2; 100)	97.8 (88.5; 99.9)	95.7 (85.2; 99.5)	100 (92.0; 100)	97.8 (88.5; 99.9)	95.6 (84.9; 99.5)	97.8 (88.2; 99.9)
	POST	100 (92.7; 100)	100 (92.1; 100)	100 (90.3; 100)	97.8 (88.2; 99.9)	95.8 (85.7; 99.5)	100 (92.3; 100)	97.7 (88.0; 99.9)	100 (92.1; 100)	100 (91.4; 100)
**23F**	PRE	85.4 (72.2; 93.9)	97.7 (87.7; 99.9)	82.9 (67.9; 92.8)	83.0 (69.2; 92.4)	84.8 (71.1; 93.7)	91.1 (78.8; 97.5)	87.0 (73.7; 95.1)	93.5 (82.1; 98.6)	84.4 (70.5; 93.5)
	POST	97.9 (88.9; 99.9)	97.8 (88.2; 99.9)	94.3 (80.8; 99.3)	97.7 (88.0; 99.9)	91.5 (79.6; 97.6)	97.8 (88.5; 99.9)	97.7 (87.7; 99.9)	97.8 (88.2; 99.9)	95.1 (83.5; 99.4)
Vaccine-related serotypes:										
**6A**	PRE	65.2 (49.8; 78.6)	46.5 (31.2; 62.3)	54.8 (38.7; 70.2)	57.4 (42.2; 71.7)	63.8 (48.5; 77.3)	68.9 (53.4; 81.8)	60.9 (45.4; 74.9)	66.7 (51.0; 80.0)	71.1 (55.7; 83.6)
	POST	93.9 (83.1; 98.7)	80.4 (66.1; 90.6)	91.7 (77.5; 98.2)	88.9 (75.9; 96.3)	89.6 (77.3; 96.5)	85.1 (71.7; 93.8)	81.8 (67.3; 91.8)	87.0 (73.7; 95.1)	92.7 (80.1; 98.5)
**19A**	PRE	70.2 (55.1; 82.7)	52.3 (36.7; 67.5)	48.8 (32.9; 64.9)	60.9 (45.4; 74.9)	46.7 (31.7; 62.1)	64.4 (48.8; 78.1)	41.3 (27.0; 56.8)	57.8 (42.2; 72.3)	62.2 (46.5; 76.2)
	POST	85.7 (72.8; 94.1)	88.9 (75.9; 96.3)	80.0 (63.1; 91.6)	84.1 (69.9; 93.4)	83.0 (69.2; 92.4)	84.8 (71.1; 93.7)	79.1 (64.0; 90.0)	86.7 (73.2; 94.9)	78.0 (62.4; 89.4)
		**Percentage of children with Protein D antibody concentrations ≥ 100 EL.U/mL (95% CI)**								
	PRE	100 (92.7; 100)	97.7 (88.0; 99.9)	95.3 (84.2; 99.4)	97.9 (88.9; 99.9)	100 (92.5; 100)	95.6 (84.9; 99.5)	97.9 (88.7; 99.9)	97.8 (88.2; 99.9)	97.8 (88.5; 99.9)
	POST	100 (92.7; 100)	100 (92.1; 100)	97.6 (87.1; 99.9)	97.8 (88.5; 99.9)	100 (92.6; 100)	95.7 (85.2; 99.5)	100 (92.3; 100)	100 (92.5; 100)	100 (91.8; 100)

**Serotype**		**Antibody GMC (95% CI)**								
		**IIBU-IIBU N = 50**	**IIBU-DIBU N = 46**	**IIBU-NIBU N = 43**	**DIBU-IIBU N = 48**	**DIBU-DIBU N = 48**	**DIBU-NIBU N = 47**	**NIBU-IIBU N = 47**	**NIBU-DIBU N = 47**	**NIBU-NIBU (Control) N = 46**
Vaccine serotypes (µg/mL)										
**1**	PRE	0.41 (0.31; 0.54)	0.36 (0.28; 0.46)	0.27 (0.22; 0.34)	0.38 (0.28; 0.53)	0.42 (0.31; 0.57)	0.34 (0.27; 0.42)	0.44 (0.34; 0.58)	0.38 (0.30; 0.48)	0.43 (0.32; 0.58)
	POST	2.87 (2.10; 3.91)	2.23 (1.57; 3.17)	2.39 (1.68; 3.41)	2.69 (1.99; 3.63)	2.44 (1.78; 3.33)	1.87 (1.36; 2.57)	2.84 (2.02; 3.99)	3.04 (2.38; 3.88)	2.84 (2.10; 3.85)
**4**	PRE	0.71 (0.55; 0.92)	0.63 (0.50; 0.80)	0.55 (0.44; 0.69)	0.63 (0.47; 0.85)	0.72 (0.55; 0.95)	0.64 (0.49; 0.83)	0.72 (0.55; 0.93)	0.73 (0.55; 0.98)	0.62 (0.47; 0.82)
	POST	4.09 (3.20; 5.22)	3.65 (2.76; 4.84)	3.22 (2.36; 4.39)	4.05 (2.99; 5.50)	3.63 (2.84; 4.62)	3.41 (2.46; 4.71)	4.04 (3.05; 5.36)	4.08 (3.11; 5.36)	4.07 (3.09; 5.35)

**5**	PRE	0.98 (0.74; 1.29)	0.83 (0.66; 1.05)	0.76 (0.59; 0.98)	0.78 (0.61; 1.00)	0.76 (0.58; 0.99)	0.70 (0.57; 0.86)	0.83 (0.66; 1.05)	0.84 (0.67; 1.04)	0.96 (0.76; 1.23)
	POST	4.50 (3.45; 5.88)	3.90 (2.95; 5.16)	4.11 (2.91; 5.81)	3.42 (2.62; 4.46)	3.33 (2.56; 4.33)	3.37 (2.46; 4.60)	4.21 (3.03; 5.86)	3.92 (3.06; 5.01)	4.48 (3.48; 5.77)
**6B**	PRE	0.63 (0.48; 0.82)	0.44 (0.31; 0.61)	0.54 (0.40; 0.72)	0.55 (0.40; 0.77)	0.62 (0.48; 0.80)	0.60 (0.45; 0.80)	0.56 (0.42; 0.75)	0.58 (0.43; 0.78)	0.67 (0.51; 0.87)
	POST	2.75 (2.16; 3.48)	1.97 (1.41; 2.74)	2.43 (1.85; 3.19)	2.13 (1.64; 2.76)	2.18 (1.69; 2.81)	1.52 (0.98; 2.34)	2.16 (1.47; 3.18)	2.32 (1.60; 3.36)	2.51 (1.96; 3.22)
**7F**	PRE	1.33 (1.07; 1.64)	0.96 (0.75; 1.23)	0.89 (0.74; 1.07)	1.08 (0.80; 1.46)	1.10 (0.90; 1.35)	1.09 (0.89; 1.34)	1.07 (0.89; 1.29)	1.31 (1.02; 1.67)	1.11 (0.83; 1.49)
	POST	5.75 (4.59; 7.21)	4.20 (3.33; 5.31)	4.20 (3.24; 5.46)	4.96 (3.77; 6.54)	4.36 (3.35; 5.66)	3.93 (3.04; 5.08)	5.43 (4.13; 7.14)	5.55 (4.38; 7.04)	4.93 (3.64; 6.69)
**9V**	PRE	1.07 (0.82; 1.39)	0.83 (0.67; 1.02)	0.83 (0.67; 1.03)	1.03 (0.79; 1.36)	1.03 (0.81; 1.30)	0.93 (0.76; 1.14)	1.09 (0.83; 1.44)	0.92 (0.76; 1.12)	1.09 (0.82; 1.46)
	POST	4.46 (3.35; 5.93)	3.30 (2.47; 4.41)	3.94 (2.80; 5.54)	3.47 (2.76; 4.36)	3.16 (2.39; 4.17)	3.07 (2.32; 4.07)	4.07 (3.00; 5.52)	3.88 (3.09; 4.87)	4.05 (3.18; 5.15)
**14**	PRE	1.76 (1.23; 2.51)	1.74 (1.25; 2.41)	1.06 (0.71; 1.57)	1.31 (0.99; 1.74)	1.70 (1.18; 2.44)	1.73 (1.29; 2.33)	1.60 (1.06; 2.39)	1.93 (1.47; 2.53)	1.95 (1.28; 2.95)
	POST	6.02 (4.47; 8.12)	5.62 (3.99; 7.92)	5.16 (3.61; 7.36)	4.54 (3.45; 5.98)	5.08 (3.80; 6.80)	4.61 (3.37; 6.30)	6.03 (4.18; 8.70)	6.56 (5.06; 8.51)	6.30 (4.45; 8.92)
**18C**	PRE	1.13 (0.88; 1.47)	1.03 (0.81; 1.32)	0.96 (0.73; 1.26)	1.08 (0.82; 1.41)	1.10 (0.86; 1.39)	1.03 (0.80; 1.31)	1.12 (0.87; 1.43)	1.39 (1.08; 1.79)	1.23 (0.93; 1.63)
	POST	9.35 (7.13; 12.25)	8.06 (5.93; 10.96)	7.78 (5.53; 10.93)	8.23 (6.37; 10.65)	7.16 (5.41; 9.46)	7.10 (5.33; 9.46)	7.15 (5.13; 9.96)	11.29 (7.96; 16.00)	8.68 (6.41; 11.75)
**19F**	PRE	1.94 (1.46; 2.57)	1.66 (1.28; 2.17)	1.72 (1.23; 2.39)	1.58 (1.14; 2.19)	1.54 (1.09; 2.19)	1.38 (1.09; 1.75)	1.25 (0.96; 1.63)	1.70 (1.27; 2.26)	1.90 (1.38; 2.62)
	POST	6.90 (4.89; 9.72)	6.64 (5.17; 8.54)	6.91 (4.88; 9.77)	5.35 (3.84; 7.47)	5.27 (3.64; 7.64)	5.57 (4.14; 7.51)	5.24 (3.66; 7.49)	7.26 (5.28; 9.97)	7.34 (5.55; 9.73)
**23F**	PRE	0.65 (0.45; 0.94)	0.64 (0.52; 0.79)	0.41 (0.28; 0.58)	0.55 (0.39; 0.77)	0.50 (0.34; 0.72)	0.65 (0.50; 0.84)	0.61 (0.45; 0.81)	0.69 (0.51; 0.92)	0.58 (0.43; 0.79)
	POST	3.72 (2.60; 5.33)	3.08 (2.25; 4.21)	2.59 (1.63; 4.11)	2.93 (2.05; 4.19)	2.16 (1.40; 3.33)	2.74 (2.06; 3.65)	3.12 (2.17; 4.48)	3.17 (2.34; 4.31)	3.33 (2.36; 4.70)
Vaccine-related serotypes (μg/mL)										
**6A**	PRE	0.31 (0.21; 0.46)	0.18 (0.12; 0.27)	0.25 (0.17; 0.37)	0.26 (0.18; 0.39)	0.32 (0.23; 0.43)	0.32 (0.21; 0.49)	0.26 (0.17; 0.40)	0.28 (0.19; 0.43)	0.34 (0.24; 0.49)
	POST	1.36 (0.93; 1.97)	0.86 (0.56; 1.34)	1.14 (0.72; 1.79)	0.89 (0.57; 1.39)	1.09 (0.78; 1.54)	0.74 (0.49; 1.12)	1.03 (0.61; 1.74)	0.99 (0.66; 1.48)	1.40 (0.97; 2.01)
**19A**	PRE	0.28 (0.19; 0.42)	0.23 (0.16; 0.32)	0.22 (0.15; 0.32)	0.24 (0.16; 0.36)	0.20 (0.14; 0.30)	0.24 (0.17; 0.33)	0.15 (0.11; 0.20)	0.20 (0.14; 0.30)	0.28 (0.19; 0.40)
	POST	1.11 (0.67; 1.83)	1.10 (0.78; 1.56)	1.05 (0.66; 1.69)	0.75 (0.48; 1.15)	0.66 (0.40; 1.10)	0.95 (0.61; 1.48)	0.67 (0.43; 1.04)	0.93 (0.58; 1.47)	0.97 (0.61; 1.53)
Protein D (EL.U/mL)										
	PRE	661.60 (517.80; 845.40)	660.40 (501.10; 870.40)	588.30 (431.80; 801.50)	622.90 (472.00; 821.90)	590.00 (456.30; 763.00)	502.00 (376.60; 669.20)	752.10 (561.20; 1008.00)	555.10 (423.10; 728.20)	777.20 (561.40; 1076.00)
	POST	2069.00 (1639.70; 2610.80)	1980.10 (1484.10; 2641.80)	1907.50 (1330.90; 2733.90)	1888.70 (1349.30; 2643.60)	1664.80 (1283.00; 2160.20)	1540.70 (1108.60; 2141.20)	2319.70 (1701.90; 3161.80)	1953.10 (1541.50; 2474.50)	2285.50 (1724.10; 3029.70)

Footnote: PHiD-CV and DTPa-HBV-IPV/Hib at 12–15 months of age, with the following prophylactic antipyretic regimen: at primary vaccination: immediate ibuprofen, and at booster: immediate (IIBU-IIBU), delayed (IIBU-DIBU) or no ibuprofen (IIBU-NIBU); at primary vaccination: delayed ibuprofen, and at booster: immediate (DIBU-IIBU), delayed (DIBU-DIBU) or no ibuprofen (DIBU-NIBU); at primary vaccination: no ibuprofen, and at booster: immediate (NIBU-IIBU), delayed (NIBU-DIBU) or no ibuprofen (NIBU-NIBU); IBU, ibuprofen.

CI, confidence interval; GMC, geometric mean antibody concentration; EL.U/mL, ELISA units/milliliter; ATP, according-to-protocol; PRE, prior to booster vaccination; POST, one month after booster dose; %, percentage; N, maximum number of children with available results.

Supplementary table 3. Pre- and post-booster antibody responses to PHiD-CV with 95% CI for the PARA groups and control group (ATP cohort for immunogenicity)

Serotype		Percentage of children with antibody concentrations ≥ 0.2 µg/mL (95% CI)			
		IPARA-NPARA N = 50	DPARA-IPARA N = 48	NPARA-IPARA N = 48	NIBU-NIBU (Control) N = 46
Vaccine serotypes:					
**1**	PRE	62.5 (47.4; 76.0)	73.2 (57.1; 85.8)	81.8 (67.3; 91.8)	80.0 (65.4; 90.4)
	POST	95.7 (85.5; 99.5)	100 (92.0; 100)	97.9 (88.7; 99.9)	97.6 (87.1; 99.9)
**4**	PRE	91.5 (79.6; 97.6)	88.9 (75.9; 96.3)	95.6 (84.9; 99.5)	88.9 (75.9; 96.3)
	POST	100 (92.5; 100)	100 (92.1; 100)	100 (92.5; 100)	100 (91.4; 100)
**5**	PRE	95.7 (85.5; 99.5)	91.1 (78.8; 97.5)	90.9 (78.3; 97.5)	100 (92.1; 100)
	POST	100 (92.5; 100)	100 (92.1; 100)	97.9 (88.7; 99.9)	100 (91.4; 100)
**6B**	PRE	88.0 (75.7; 95.5)	87.2 (74.3; 95.2)	93.3 (81.7; 98.6)	95.6 (84.9; 99.5)
	POST	91.7 (80.0; 97.7)	97.8 (88.5; 99.9)	100 (92.5; 100)	100 (91.6; 100)
**7F**	PRE	98.0 (89.1; 99.9)	97.7 (87.7; 99.9)	95.7 (85.2; 99.5)	97.7 (88.0; 99.9)
	POST	100 (92.5; 100)	100 (92.1; 100)	97.9 (88.7; 99.9)	100 (91.4; 100)
**9V**	PRE	98.0 (89.4; 99.9)	100 (92.5; 100)	95.8 (85.7; 99.5)	100 (92.1; 100)
	POST	100 (92.6; 100)	100 (92.3; 100)	97.9 (88.9; 99.9)	100 (91.6; 100)
**14**	PRE	95.9 (86.0; 99.5)	97.7 (88.0; 99.9)	97.8 (88.5; 99.9)	97.8 (88.2; 99.9)
	POST	100 (92.9; 100)	100 (92.5; 100)	100 (92.5; 100)	100 (91.8; 100)
**18C**	PRE	96.0 (86.3; 99.5)	100 (92.5; 100)	100 (92.5; 100)	95.7 (85.2; 99.5)
	POST	100 (92.7; 100)	100 (92.0; 100)	100 (92.5; 100)	100 (91.6; 100)
**19F**	PRE	97.9 (88.9; 99.9)	97.8 (88.2; 99.9)	95.6 (84.9; 99.5)	97.8 (88.2; 99.9)
	POST	100 (92.5; 100)	97.7 (88.0; 99.9)	97.9 (88.7; 99.9)	100 (91.4; 100)
**23F**	PRE	83.7 (70.3; 92.7)	80.0 (65.4; 90.4)	87.0 (73.7; 95.1)	84.4 (70.5; 93.5)
	POST	95.7 (85.5; 99.5)	93.2 (81.3; 98.6)	97.9 (88.7; 99.9)	95.1 (83.5; 99.4)
Vaccine-related serotypes:					
**6A**	PRE	45.8 (31.4; 60.8)	48.9 (33.7; 64.2)	59.1 (43.2; 73.7)	71.1 (55.7; 83.6)
	POST	83.7 (70.3; 92.7)	87.2 (74.3; 95.2)	83.0 (69.2; 92.4)	92.7 (80.1; 98.5)
**19A**	PRE	60.4 (45.3; 74.2)	50.0 (34.6; 65.4)	57.8 (42.2; 72.3)	62.2 (46.5; 76.2)
	POST	83.0 (69.2; 92.4)	77.3 (62.2; 88.5)	87.2 (74.3; 95.2)	78.0 (62.4; 89.4)
		**Percentage of children with Protein D antibody concentrations ≥ 100 EL.U/mL (95% CI)**			
	PRE	92.0 (80.8; 97.8)	93.5 (82.1; 98.6)	100 (92.6; 100)	
	POST	98.0 (89.4; 99.9)	100 (92.6; 100)	100 (92.6; 100)	

Serotype		Antibody GMC (95% CI)			
		IPARA-NPARA N = 50	DPARA-IPARA N = 48	NPARA-IPARA N = 48	NIBU-NIBU (Control) N = 46
Vaccine serotypes (µg/mL)					
**1**	PRE	0.31 (0.24; 0.40)	0.30 (0.23; 0.40)	0.45 (0.32; 0.64)	0.43 (0.32; 0.58)
	POST	1.76 (1.29; 2.38)	2.14 (1.59; 2.89)	2.84 (2.06; 3.89)	2.84 (2.10; 3.85)
**4**	PRE	0.52 (0.39; 0.68)	0.60 (0.48; 0.76)	0.84 (0.64; 1.11)	0.62 (0.47; 0.82)
	POST	3.27 (2.51; 4.25)	3.31 (2.66; 4.12)	4.28 (3.23; 5.68)	4.07 (3.09; 5.35)
**5**	PRE	0.59 (0.45; 0.77)	0.72 (0.57; 0.91)	0.85 (0.62; 1.17)	0.96 (0.76; 1.23)
	POST	2.62 (1.92; 3.57)	3.58 (2.74; 4.69)	4.33 (3.26; 5.76)	4.48 (3.48; 5.77)
**6B**	PRE	0.43 (0.33; 0.56)	0.43 (0.33; 0.57)	0.61 (0.49; 0.76)	0.67 (0.51; 0.87)
	POST	1.74 (1.23; 2.45)	1.84 (1.36; 2.49)	2.29 (1.79; 2.91)	2.51 (1.96; 3.22)
**7F**	PRE	0.84 (0.67; 1.06)	0.98 (0.79; 1.22)	0.97 (0.76; 1.24)	1.11 (0.83; 1.49)
	POST	3.89 (2.98; 5.10)	4.63 (3.56; 6.04)	4.52 (3.31; 6.16)	4.93 (3.64; 6.69)
**9V**	PRE	0.74 (0.58; 0.95)	0.93 (0.76; 1.14)	0.97 (0.73; 1.28)	1.09 (0.82; 1.46)
	POST	3.11 (2.36; 4.09)	3.49 (2.68; 4.56)	3.90 (2.86; 5.30)	4.05 (3.18; 5.15)
**14**	PRE	1.18 (0.85; 1.64)	1.53 (1.08; 2.16)	1.86 (1.38; 2.52)	1.95 (1.28; 2.95)
	POST	4.72 (3.56; 6.27)	5.52 (4.13; 7.38)	5.62 (4.23; 7.48)	6.30 (4.45; 8.92)
**18C**	PRE	0.91 (0.67; 1.23)	1.14 (0.92; 1.41)	1.31 (1.01; 1.69)	1.23 (0.93; 1.63)
	POST	6.18 (4.65; 8.20)	8.66 (6.76; 11.09)	8.17 (5.80; 11.52)	8.68 (6.41; 11.75)
**19F**	PRE	1.56 (1.14; 2.13)	1.51 (1.15; 2.00)	1.72 (1.31; 2.27)	1.90 (1.38; 2.62)
	POST	5.94 (4.43; 7.97)	5.54 (3.88; 7.93)	6.66 (4.92; 9.01)	7.34 (5.55; 9.73)
**23F**	PRE	0.47 (0.34; 0.65)	0.43 (0.30; 0.62)	0.52 (0.37; 0.74)	0.58 (0.43; 0.79)
	POST	2.50 (1.76; 3.56)	2.53 (1.74; 3.68)	3.15 (2.31; 4.30)	3.33 (2.36; 4.70)

Vaccine-related serotypes (µg/mL)					
**6A**	PRE	0.19 (0.13; 0.27)	0.22 (0.16; 0.31)	0.24 (0.17; 0.35)	0.34 (0.24; 0.49)
	POST	0.81 (0.53; 1.23)	0.87 (0.59; 1.29)	0.98 (0.67; 1.43)	1.40 (0.97; 2.01)
**19A**	PRE	0.21 (0.l5; 0.30)	0.19 (0.13; 0.28)	0.24 (0.16; 0.36)	0.28 (0.19; 0.40)
	POST	0.74 (0.49; 1.10)	0.63 (0.37; 1.09)	1.11 (0.71; 1.74)	0.97 (0.61; 1.53)
Protein D (EL.U/mL)					
	PRE	446.1 (331.1; 601.0)	525.4 (367.8; 750.4)	691.3 (550.1; 868.8)	777.20 (561.40; 1076.00)
	POST	1482.7 (1077.1; 2040.9)	1517.3 (1134.5; 2029.2)	2082.5 (1634.9; 2652.5)	2285.50 (1724.10; 3029.70)

Footnote: PHiD-CV and DTPa-HBV-IPV/Hib at 12–15 months of age, with the following prophylactic antipyretic regimen: no ibuprofen at primary vaccination, and no ibuprofen at booster (NIBU-NIBU); immediate paracetamol at primary vaccination and no paracetamol at booster (IPARA-NPARA); delayed paracetamol at primary vaccination and immediate paracetamol at booster (DPARA-IPARA); No paracetamol at primary vaccination, and immediate paracetamol at booster (NPARA-IPARA).

IBU, ibuprofen; PARA, paracetamol; CI, confidence interval; GMC, geometric mean antibody concentration; EL.U/mL, ELISA units/milliliter; ATP, according-to-protocol; PRE, prior to booster vaccination; POST, one month after booster dose; %, percentage; N, maximum number of children with available results.

Supplementary table 4. Pre- and post-booster antibody responses to DTPa-HBV-IPV/Hib antigens with 95% CI for the IBU groups (ATP cohort for immunogenicity)

Antibody (cut-off/threshold)		Seroprotection/seropositivity rates (95% CI)								
		**IIBU-IIBU N = 48**	**IIBU-DIBU N = 45**	**IIBU-NIBU N = 40**	**DIBU-IIBU N = 46**	**DIBU-DIBU N = 47**	**DIBU-NIBU N = 45**	**NIBU-IIBU N = 45**	**NIBU-DIBU N = 45**	**NIBU-NIBU (Control) N = 44**
**DIPHT (≥ 0.1 IU/mL)**	PRE	97.8 (88.2; 99.9)	100 (91.4; 100)	100 (91.2; 100)	97.8 (88.5; 99.9)	100 (92.0; 100)	97.7 (87.7; 99.9)	100 (91.8; 100)	100 (91.4; 100)	97.7 (87.7; 99.9)
	POST	100 (92.6; 100)	100 (92.0; 100)	100 (89.7; 100)	100 (92.0; 100)	100 (92.3; 100)	100 (92.1; 100)	100 (91.6; 100)	100 (92.1; 100)	100 (91.2; 100)
**TET (≥ 0.1 IU/mL)**	PRE	100 (92.0; 100)	97.5 (86.8; 99.9)	100 (91.0; 100)	97.8 (88.5; 99.9)	100 (92.0; 100)	100 (91.8; 100)	97.7 (87.7; 99.9)	100 (91.4; 100)	100 (91.8; 100)
	POST	100 (92.6; 100)	100 (92.0; 100)	100 (89.7; 100)	100 (92.0; 100)	100 (92.3; 100)	100 (92.1; 100)	100 (91.6; 100)	100 (92.1; 100)	100 (91.2; 100)
**PT (≥ 5 EL.U/mL)**	PRE	93.2 (81.3; 98.6)	100 (91.2; 100)	89.2 (74.6; 97.0)	88.6 (75.4; 96.2)	100 (91.8; 100)	95.3 (84.2; 99.4)	88.4 (74.9; 96.1)	84.6 (69.5; 94.1)	97.7 (87.7; 99.9)
	POST	100 (92.6; 100)	100 (92.0; 100)	94.1 (80.3; 99.3)	100 (92.0; 100)	100 (92.3; 100)	97.7 (87.7; 99.9)	97.6 (87.1; 99.9)	100 (92.0; 100)	100 (91.2; 100)
**FHA (≥ 5 EL.U/mL)**	PRE	100 (92.0; 100)	100 (91.2; 100)	100 (90.5; 100)	100 (92.0; 100)	100 (91.8; 100)	100 (91.0; 100)	100 (91.8; 100)	100 (90.7; 100)	100 (91.6; 100)
	POST	100 (92.6; 100)	100 (92.0; 100)	100 (89.7; 100)	100 (92.0; 100)	100 (92.3; 100)	100 (91.8; 100)	100 (91.4; 100)	100 (92.0; 100)	100 (91.2; 100)
**PRN (≥ 5 EL.U/mL)**	PRE	87.2 (74.3; 95.2)	95.1 (83.5; 99.4)	95.0 (83.1; 99.4)	95.7 (85.2; 99.5)	97.7 (88.0; 99.9)	86.4 (72.6; 94.8)	86.7 (73.2; 94.9)	93.3 (81.7; 98.6)	100 (92.0; 100)
	POST	100 (92.6; 100)	100 (92.1; 100)	97.1 (84.7; 99.9)	100 (92.0; 100)	100 (92.3; 100)	95.6 (84.9; 99.5)	100 (91.6; 100)	100 (92.1; 100)	100 (91.2; 100)
**PRP (≥ 0.15 µg/mL)**	PRE	93.5 (82.1; 98.6)	90.2 (76.9; 97.3)	85.0 (70.2; 94.3)	91.3 (79.2; 97.6)	95.5 (84.5; 99.4)	88.6 (75.4; 96.2)	88.9 (75.9; 96.3)	97.7 (87.7; 99.9)	100 (92.0; 100)
	POST	100 (92.6; 100)	100 (92.1; 100)	100 (89.7; 100)	100 (92.0; 100)	100 (92.5; 100)	100 (92.1; 100)	100 (91.4; 100)	100 (92.1; 100)	100 (91.4; 100)
**HBs (≥ 10 mIU/mL)**	PRE	100 (89.7; 100)	94.3 (80.8; 99.3)	96.7 (82.8; 99.9)	94.1 (80.3; 99.3)	97.1 (85.1; 99.9)	100 (90.0; 100)	97.1 (84.7; 99.9)	96.7 (82.8; 99.9)	94.4 (81.3; 99.3)
	POST	100 (91.8; 100)	100 (91.0; 100)	100 (88.1; 100)	97.6 (87.1; 99.9)	100 (90.7; 100)	100 (91.2; 100)	100 (89.4; 100)	100 (90.5; 100)	100 (91.2; 100)

Antibody (cut-off)		Antibody GMC (95% CI)								
		**IIBU-IIBU N = 48**	**IIBU-DIBU N = 45**	**IIBU-NIBU N = 40**	**DIBU-IIBU N = 46**	**DIBU-DIBU N = 47**	**DIBU-NIBU N = 45**	**NIBU-IIBU N = 45**	**NIBU-DIBU N = 45**	**NIBU-NIBU (Control) N = 44**
**DIPHT (≥ 0.1 IU/mL)**	PRE	0.736 (0.556; 0.975)	0.593 (0.476; 0.739)	0.616 (0.499; 0.762)	0.550 (0.439; 0.690)	0.581 (0.428; 0.788)	0.628 (0.483; 0.815)	0.714 (0.581; 0.876)	0.627 (0.509; 0.774)	0.656 (0.514; 0.836)
	POST	7.492 (5.932; 9.463)	5.257 (4.033; 6.853)	7.570 (5.512; 10.396)	5.926 (4.752; 7.390)	5.831 (4.642; 7.324)	6.486 (4.778; 8.805)	7.059 (5.455; 9.136)	7.226 (5.872; 8.892)	8.206 (6.869; 9.802)
**TET (≥ 0.1 IU/mL)**	PRE	0.838 (0.661; 1.062)	0.735 (0.573; 0.944)	0.660 (0.519; 0.839)	0.681 (0.538; 0.862)	0.868 (0.668; 1.130)	0.692 (0.531; 0.901)	0.843 (0.661; 1.076)	0.883 (0.686; 1.136)	0.858 (0.671; 1.097)
	POST	8.030 (6.347; 10.159)	6.887 (5.372; 8.829)	7.283 (5.179; 10.243)	7.092 (5.734; 8.772)	7.269 (5.916; 8.932)	6.522 (4.886; 8.707)	7.095 (5.320; 9.461)	10.800 (8.799; 13.254)	9.045 (7.659; 10.681)
**PT (≥ 5 EL.U/mL)**	PRE	13.3 (10.8; 16.3)	12.8 (11.0; 14.9)	10.5 (8.2; 13.3)	14.7 (11.1; 19.6)	14.2 (11.4; 17.8)	13.6 (11.1; 16.7)	12.8 (9.9; 16.7)	12.0 (9.1; 15.9)	15.2 (12.4; 18.5)
	POST	73.8 (58.9; 92.5)	64.4 (50.4; 82.3)	56.6 (39.9; 80.5)	72.3 (57.7; 90.6)	75.5 (59.4; 95.9)	74.8 (56.9; 98.2)	63.4 (48.0; 83.6)	74.9 (60.0; 93.4)	97.6 (82.5; 115.5)
**FHA (≥ 5 EL.U/mL)**	PRE	46.1 (36.7; 57.9)	42.6 (34.5; 52.6)	42.6 (33.3; 54.5)	53.8 (40.2; 72.2)	59.3 (44.1; 79.8)	57.7 (41.7; 79.9)	57.9 (42.5; 78.8)	45.7 (36.3; 57.6)	59.1 (46.4; 75.1)
	POST	308.8 (247.1; 385.9)	252.7 (195.1; 327.4)	327.2 (240.9; 444.4)	359.8 (289.2; 447.6)	322.9 (252.2; 413.4)	332.7 (250.9; 441.2)	312.3 (238.7; 408.8)	338.6 (270.3; 424.3)	442.8 (370.6; 529)
**PRN (≥ 5 EL.U/mL)**	PRE	15.7 (11.8; 21.1)	16.0 (12.9; 19.9)	18.3 (13.8; 24.4)	22.4 (16.5; 30.5)	25.1 (18.5; 34.0)	16.0 (11.6; 22.1)	19.4 (13.5; 27.9)	18.8 (14.1; 24.9)	27.3 (20.9; 35.7)
	POST	218.6 (162.4; 294.3)	173.5 (131.0; 229.8)	225.9 (145.9; 349.8)	262.9 (196.5; 351.7)	246.2 (182.5; 332.0)	184.3 (122.6; 277.1)	226.7 (162.6; 316.1)	255.7 (189.9; 344.3)	330.4 (263.9; 413.7)
**PRP (≥ 0.15 µg/mL)**	PRE	0.878 (0.609; 1.266)	0.684 (0.480; 0.974)	0.678 (0.436; 1.055)	0.824 (0.554; 1.226)	0.847 (0.571; 1.256)	0.763 (0.510; 1.141)	0.798 (0.518; 1.231)	0.720 (0.531; 0.977)	1.013 (0.689; 1.491)
	POST	21.964 (15.760; 30.610)	17.484 (11.592; 26.370)	21.277 (12.268; 36.901)	20.280 (13.960; 29.462)	18.987 (13.365; 26.973)	17.544 (11.966; 25.722)	20.659 (14.948; 28.550)	33.450 (23.717; 47.176)	22.083 (14.795; 32.961)

**HBs (≥ 10 mIU/mL)**	PRE	197.46 (125.70; 310.18)	210.32 (125.47; 352.56)	164.33 (98.38; 274.49)	136.53 (77.08; 241.83)	225.01 (135.08; 374.83)	194.51 (128.13; 295.29)	226.10 (134.40; 380.39)	244.52 (133.36; 448.35)	159.79 (96.78; 263.80)
	POST	1949.42 (1220.13; 3114.61)	1898.54 (1147.47; 3141.21)	1970.60 (1017.24; 3817.44)	1685.87 (1012.93; 2805.87)	2492.42 (1638.97; 3790.27)	2107.75 (1257.97; 3531.56)	1851.22 (1033.84; 3314.85)	2579.59 (1575.91; 4222.52)	3244.33 (2235.34; 4708.75)

Footnote: DIPHT, Diphtheria; TET, Tetanus; PT, Pertussis Toxoid; FHA, Filamentous Haemagglutinin; PRN, Pertactin; PRP, Polyribosyl-ribitol Phosphate; HBs, Hepatitis B Surface; EL.U/mL, ELISA units/milliliter; mIU/mL, milli-international units/milliliter; PHiD-CV and DTPa-HBV-IPV/Hib at 12–15 months of age, with the following prophylactic antipyretic regimen: at primary vaccination: immediate ibuprofen, and at booster: immediate (IIBU-IIBU), delayed (IIBU-DIBU) or no ibuprofen (IIBU-NIBU); at primary vaccination: delayed ibuprofen, and at booster: immediate (DIBU-IIBU), delayed (DIBU-DIBU) or no ibuprofen (DIBU-NIBU); at primary vaccination: no ibuprofen, and at booster: immediate (NIBU-IIBU), delayed (NIBU-DIBU) or no ibuprofen (NIBU-NIBU). IBU, ibuprofen; CI, confidence interval; GMC, geometric mean concentration; ATP, according-to-protocol; PRE, prior to booster vaccination; POST, one month after booster dose; N, maximum number of children with available results.

Supplementary table 5. Pre- and post-booster antibody responses to DTPa-HBV-IPV/Hib antigens with 95% CI for the PARA groups (ATP cohort for immunogenicity)

Antibody (cut-off/threshold)		Seroprotection/seropositivity rates (95% CI)			Antibody GMC (95% CI)		
		IPARA-NPARA N = 49	DPARA-IPARA N = 44	NPARA-IPARA N = 47	IPARA-NPARA N = 49	DPARA-IPARA N = 44	NPARA-IPARA N = 47
**DIPHT (≥ 0.1 IU/mL)**	PRE	100 (92.3; 100)	95.1 (83.5; 99.4)	100 (92.0; 100)	0.665 (0.552; 0.800)	0.546 (0.417; 0.715)	0.642 (0.518; 0.795)
	POST	100 (92.5; 100)	100 (92.0; 100)	97.9 (88.7; 99.9)	7.238 (5.322; 9.845)	6.477 (5.069; 8.277)	6.749 (4.931; 9.237)
**TET (≥ 0.1 IU/mL)**	PRE	97.8 (88.5; 99.9)	97.5 (86.8; 99.9)	100 (92.0; 100)	0.666 (0.523; 0.849)	0.698 (0.538; 0.907)	0.900 (0.707; 1.146)
	POST	100 (92.5; 100)	100 (92.0; 100)	100 (92.3; 100)	6.491 (4.930; 8.546)	7.431 (5.672; 9.736)	7.423 (5.625; 9.796)
**PT (≥ 5 EL.U/mL)**	PRE	93.3 (81.7; 98.6)	86.8 (71.9; 95.6)	88.6 (75.4; 96.2)	13.0 (10.6; 16.1)	10.8 (8.6; 13.7)	14.2 (10.5; 19.1)
	POST	100 (92.3; 100)	97.7 (88.0; 99.9)	95.5 (84.5; 99.4)	59.4 (46.8; 75.2)	66.8 (50.6; 88.3)	57.0 (42.2; 77.1)
**FHA (≥ 5 EL.U/mL)**	PRE	100 (92.1; 100)	100 (90.7; 100)	100 (91.8; 100)	50.8 (42.1; 61.3)	48.5 (39.7; 59.3)	52.9 (39.1; 71.7)
	POST	100 (92.3; 100)	100 (92.0; 100)	100 (92.0; 100)	321.0 (246.5; 417.9)	332.9 (258.2; 429.3)	294.2 (224.3; 385.9)
**PRN (≥ 5 EL.U/mL)**	PRE	98.0 (89.1; 99.9)	95.2 (83.8; 99.4)	91.1 (78.8; 97.5)	20.1 (14.7; 27.6)	19.7 (14.6; 26.7)	20.3 (14.3; 29.0)
	POST	100 (92.7; 100)	100 (92.0; 100)	97.9 (88.7; 99.9)	205.1 (148.5; 283.5)	214.2 (151.8; 302.3)	213.6 (151.2; 301.8)
**PRP (≥ 0.15 µg/mL)**	PRE	97.8 (88.5; 99.9)	90.5 (77.4; 97.3)	95.5 (84.5; 99.4)	0.696 (0.500; 0.967)	0.651 (0.452; 0.938)	0.953 (0.655; 1.386)
	POST	100 (92.5; 100)	100 (92.0; 100)	100 (92.5; 100)	16.682 (11.367; 24.481)	21.602 (14.406; 32.392)	23.277 (16.047; 33.765)
**HBs (≥ 10 mIU/mL)**	PRE	93.9 (79.8; 99.3)	97.0 (84.2; 99.9)	93.9 (79.8; 99.3)	128.12 (78.68; 208.60)	199.57 (115.04; 346.22)	209.27 (121.25; 361.16)
	POST	100 (90.5; 100)	97.3 (85.8; 99.9)	100 (90.3; 100)	2078.63 (1261.91; 3423.92)	2003.09 (1040.72; 3855.37)	2218.23 (1219.17; 4035.97)

Footnote: DIPHT, Diphtheria; TET, Tetanus; PT, Pertussis Toxoid; FHA, Filamentous Haemagglutinin; PRN, Pertactin; PRP, Polyribosyl-ribitol Phosphate; HBs, Hepatitis B Surface; EL.U/mL, ELISA units/milliliter; mIU/mL, milli-international units/milliliter; PHiD-CV and DTPa-HBV-IPV/Hib at 12–15 months of age, with the following prophylactic antipyretic regimen: immediate paracetamol at primary vaccination and no paracetamol at booster vaccination (IPARA-NPARA), delayed paracetamol at primary vaccination and immediate paracetamol at booster vaccination (DPARA-IPARA) or no paracetamol at primary vaccination and immediate paracetamol at booster vaccination (NPARA-IPARA).

CI, confidence interval; GMC, geometric mean concentration; ATP, according-to-protocol; PRE, prior to booster vaccination; POST, one month after booster dose; N, maximum number of children with available results.

Supplementary table 6. Factorial design comparisons (two-way ANOVA model) for antibody GMCs, one month post-booster vaccination (ATP cohort for immunogenicity)

	Factor		
**Antibody**	**Primary ibuprofen administration (p-value)**	**Booster ibuprofen administration (p-value)**	**Interaction (primary x booster ibuprofen administration) (p-value)**
**Serotype 1**	0.1842	0.3821	0.6717
**Serotype 4**	0.5793	0.5036	0.9386
**Serotype 5**	0.0951	0.7530	0.9785
**Serotype 6B**	0.1960	0.7045	0.2586
**Serotype 7F**	0.1943	0.1231	0.7635
**Serotype 9V**	0.1158	0.4000	0.8730
**Serotype 14**	0.0833	0.8431	0.9631
**Serotype 18C**	0.3599	0.7106	0.2412
**Serotype 19F**	0.1679	0.6152	0.7453
**Serotype 23F**	0.2852	0.5173	0.6709
**Serotype 6A**	0.3324	0.8286	0.225
**Serotype 19A**	0.1722	0.6100	0.7600
**Protein D**	0.0976	0.5733	0.9228
**Diphtheria**	0.1141	0.1427	0.5339
**Tetanus**	**0.0417**	0.5771	0.1754
**Pertussis Toxoid**	0.1784	0.8035	0.1020
**Filamentous Haemagglutinin**	0.1251	0.1854	0.4297
**Pertactin**	0.1249	0.8250	0.1383
**Polyribosylribitol Phosphate**	0.1875	0.8113	0.3398
**Hepatitis B Surface**	0.4448	0.3576	0.7600

Footnote: Primary ibuprofen administration, different types of ibuprofen administration after each primary doses- 3 modalities (immediate, delayed, no); Booster ibuprofen administration, different types of ibuprofen administration after booster dose- 3 modalities (immediate, delayed, no); Interaction, interaction between the 2 previous factors; ATP, according-to-protocol; GMC, geometric mean concentration; p-value, p-value linked to the F-test.

Two way ANOVA model on the log-transformed concentration considering primary ibuprofen administration, booster ibuprofen administration and interaction as fixed effects. 2 degrees of freedom for primary or ibuprofen administration and 4 degrees of freedom for interaction. Interaction (primary x booster ibuprofen administration) is considered as statistically significant if 2-sided p-value <0.05 (marked in **bold**).
